# NRF2 activation reprogrammes defects in oxidative metabolism to restore macrophage function in COPD

**DOI:** 10.1164/rccm.202203-0482OC

**Published:** 2023-02-01

**Authors:** Eilise M. Ryan, Pranvera Sadiku, Patricia Coelho, Emily R. Watts, Ailiang Zhang, Andrew J.M. Howden, Manuel A. Sanchez-Garcia, Martin Bewley, Joby Cole, Brian J. McHugh, Wesley Vermaelen, Bart Ghesquiere, Peter Carmeliet, Giovanny Rodriguez Blanco, Alex Von Kriegsheim, Yolanda Sanchez, William Rumsey, James F. Callahan, George Cooper, Nicholas Parkinson, Kenneth Baillie, Doreen A. Cantrell, John McCafferty, Gourab Choudhury, Dave Singh, David H. Dockrell, Moira K.B. Whyte, Sarah R. Walmsley

**Affiliations:** 1 University of Edinburgh Centre for Inflammation Research, The Queen’s Medical Research Institute, University of Edinburgh, Edinburgh, UK; 2 Division of Cell Signalling and Immunology, University of Dundee, Dundee, DD1 5EH, UK; 3 Department of Infection, Immunity and Cardiovascular Disease, University of Sheffield, Sheffield, UK; 4 Metabolomics Expertise Centre, VIB-KU Leuven Centre for Cancer Biology, Leuven 3000, Belgium; 5 Laboratory of Angiogenesis and Vascular Metabolism, Centre for Cancer Biology, VIB, Department of Oncology, Leuven Cancer Institute, KU Leuven, Leuven 3000, Belgium; 6 Laboratory for Translational Breast Cancer Research, Department of Oncology, KU Leuven, Leuven 3000, Belgium; 7 State Key Laboratory of Ophthalmology, Zhongshan Ophthalmic Centre, Sun Yat-Sen University, Guangzhou, Guangdong, P.R. China; 8 Cancer Research UK Edinburgh Centre, Institute of Genetics and Cancer, University of Edinburgh, Edinburgh, UK; 9 GlaxoSmithKline Research & Development, Collegeville, PA 19426, United States; 10 MRC Human Genetics Unit, Institute of Genetics and Molecular Medicine, University of Edinburgh, Edinburgh EH4 2XU, UK; 11 NHS Lothian, Respiratory Medicine, Edinburgh, UK; 12 Division of Infection, Immunity and Respiratory Medicine, University of Manchester, Manchester, UK

**Keywords:** Chronic obstructive pulmonary disease, macrophage, metabolism, nuclear factor erythroid 2–related factor 2 (NRF2), malic enzyme 1

## Abstract

**Rationale:**

COPD (Chronic Obstructive Pulmonary Disease) is a disease characterized by persistent airway inflammation and disordered macrophage function. The extent to which alterations in macrophage bioenergetics contribute to impaired antioxidant responses and disease pathogenesis has yet to be fully delineated.

**Objectives:**

Through the study of COPD alveolar (AM) and peripheral monocyte-derived (MDM) macrophages, we sought to establish if intrinsic defects in core metabolic processes drive macrophage dysfunction and redox imbalance.

**Methods:**

AM and MDM from COPD and healthy donors underwent functional, metabolic and transcriptional profiling.

**Results:**

We observe that AM and MDM from COPD donors display a critical depletion in glycolytic and mitochondrial respiration derived energy reserves and an over reliance on glycolysis as a source for ATP, resulting in reduced energy status. Defects in oxidative metabolism extend to an impaired redox balance associated with defective expression of the NADPH generating enzyme, malic enzyme 1, a known target of the anti-oxidant transcription factor NRF2. Consequently, selective activation of NRF2 resets the COPD transcriptome, resulting in increased generation of TCA cycle intermediaries, improved energetic status, favorable redox balance and a recovery of macrophage function.

**Conclusion:**

In COPD an inherent loss of metabolic plasticity leads to metabolic exhaustion and reduced redox capacity which can be rescued by activation of the NRF2 pathway. Targeting these defects, via NRF2 augmentation, may therefore present an attractive therapeutic strategy for the treatment of the aberrant airway inflammation described in COPD.

## Introduction

COPD is the third leading cause of death globally. To date we have no therapies which significantly alter the course of this debilitating disease. The histological hallmark of COPD is persistent inflammation of the airways, resulting in airflow limitation, measured by a decline in forced expiratory volume in 1 second (FEV1), chronic bronchitis and emphysema. This inflammation persists in people with COPD, even following smoking cessation^1^.

It has long been established that macrophages play a major role in the pathogenesis of COPD. Alveolar macrophages are present in high numbers in the airways and airway secretions of patients with COPD, where their abundance correlates directly with disease severity^2^. Despite this abundance, patients with COPD experience high rates of infection, with pathogenic bacteria in the lower airways strongly associated with exacerbation frequency and increased inflammation^3^. This major disconnect, whereby excessive cellular inflammation results in ineffective immunity, is now understood to be largely driven by macrophage dysfunction. Work from our group and others has previously shown that COPD macrophages have defective phagocytosis of bacteria that colonize the lungs in COPD, such as non-typeable *Haemophilus influenzae* and *Streptococcus pneumoniae*^4,5,6,7^. Macrophage dysfunction also extends to impaired inflammation resolution, with failure to efferocytose apoptotic cells also described^8^. In conjunction with impaired macrophage internalization rates, macrophages in COPD also produce molecules known to induce pulmonary tissue damage including matrix metalloproteinases (MMPs)^9,10^ and reactive oxygen species (ROS)^11^. ROS, in particular, have been linked to mitochondrial dysfunction in COPD^12^. Thus, in COPD, macrophages demonstrate functional responses that are ineffective and skew the balance towards “self-injury” in the lung. Despite these well characterized defects in macrophage function, the mechanisms that drive this aberrant behaviour remain poorly defined^7,13,14^. Therefore, through the study of macrophages isolated from the airways [Alveolar Macrophages (AM)] and peripheral blood [Monocyte-derived macrophages (MDM)] of COPD and healthy donors we sought to delineate the intrinsic processes that promote macrophage dysfunction in COPD. We demonstrate that in COPD, alveolar and monocyte-derived macrophages share an inherent defect in efferocytosis and phagocytosis, driven by the loss of oxidative and glycolytic reserve capacity and impaired metabolic plasticity. Reduced expression of the NADPH generating enzyme malic enzyme 1, recapitulates these metabolic defects and results in a reduction in redox buffering capacity and impaired efferocytosis. By activating the anti-oxidant transcription factor NRF2, we were able to rescue ME1 expression and restore metabolic plasticity and function in COPD macrophages. Some of the results of this study have been previously reported in the form of abstracts^15,16,17,18^.

## Materials and Methods

### Macrophage donors

COPD and healthy donors underwent bronchoscopy and venesection. Patient demographics are outlined in Table 1. Written informed consent was obtained in accordance with local ethics approval, as detailed in the online supplement.

### Cell Culture

Alveolar macrophages were isolated from BAL as previously described^19^, with 93-97% purity as assessed by Cytospin. MDM were differentiated from peripheral blood mononuclear cells isolated via discontinuous plasma-Percoll gradients (Sigma-Aldrich), for 14 days in culture. For Seahorse MDM experiments, monocytes were isolated using the Miltenyi Biotec Pan-Monocyte Isolation kit then plated into low-adherence flasks (Corning) for culture. THP-1 cells were differentiated to macrophages by treating with 100 nM PMA for 3 days, then rested for 3 days prior to assay. Cells were treated with 0.065 μM KI-696, a selective inhibitor of the KEAP1–NRF2 interaction or vehicle control x16 hours prior to assays. All cells were cultured in RMPI 1640 (Sigma-Aldrich) with 10% heat inactivated FCS (Gibco).

### Functional assays

Bacterial internalisation assays were performed as previously described^7^, using opsonized Serotype 14 *S. pneumoniae* (National Collection of Type Cultures 11902). For efferocytosis assays, neutrophils isolated via Percoll-gradient were stained with a PKH26 labelling kit, as per manufacturers guidelines (Sigma-Aldrich), and cultured for 20h in RMPI 1640. Apoptotic neutrophils (70-80% apoptotic verified by Annexin-V-Topro3 staining) were added to macrophages at MOI 5:1, with ice control, for 90 minutes before vigorously washing and removal for analysis on a Becton Dickinson FACS Calibur flow cytometer^20^.

### Quantification and Statistical Analysis

Data represents mean ± SEM. Statistical parametric analyses were performed following confirmation of normal distribution of the data via D’Agostino-Pearson normality tests. All statistical tests were performed with Prism 8 software (GraphPad Software) using unpaired t-tests, paired t-tests, one-way or two-way ANOVA with Sidak’s multiple comparisons test, Pearsons ‘s correlation.

Wilcoxon matched paired signs rank test, Mann Whitney and Kruskal Wallis with Dunns multiple comparison tests were performed for nonparametric or n≤ 6 data. *P ≤0.05, **P≤0.01, ***P≤0.001.

## Results

### Correlation of defective macrophage efferocytosis with phagocytosis in COPD

To study macrophage efferocytosis and phagocytosis we recruited COPD patients with mild (14 patients, 27%) moderate (26 patients, 50%), severe (11 patients, 21%) and very severe disease (1 patient, 2%), who were free from exacerbation for ≥8 weeks minimum and age matched (± 8 years; mean age) with healthy controls (Table 1). In line with disease status, forced expiratory volume in one second (FEV_1_) was significantly lower in COPD donors and 43/52 (82%) of COPD donors had COPD Assessment test scores (CAT scores) ≥10, in keeping with severe symptoms.

**Figure 1:**
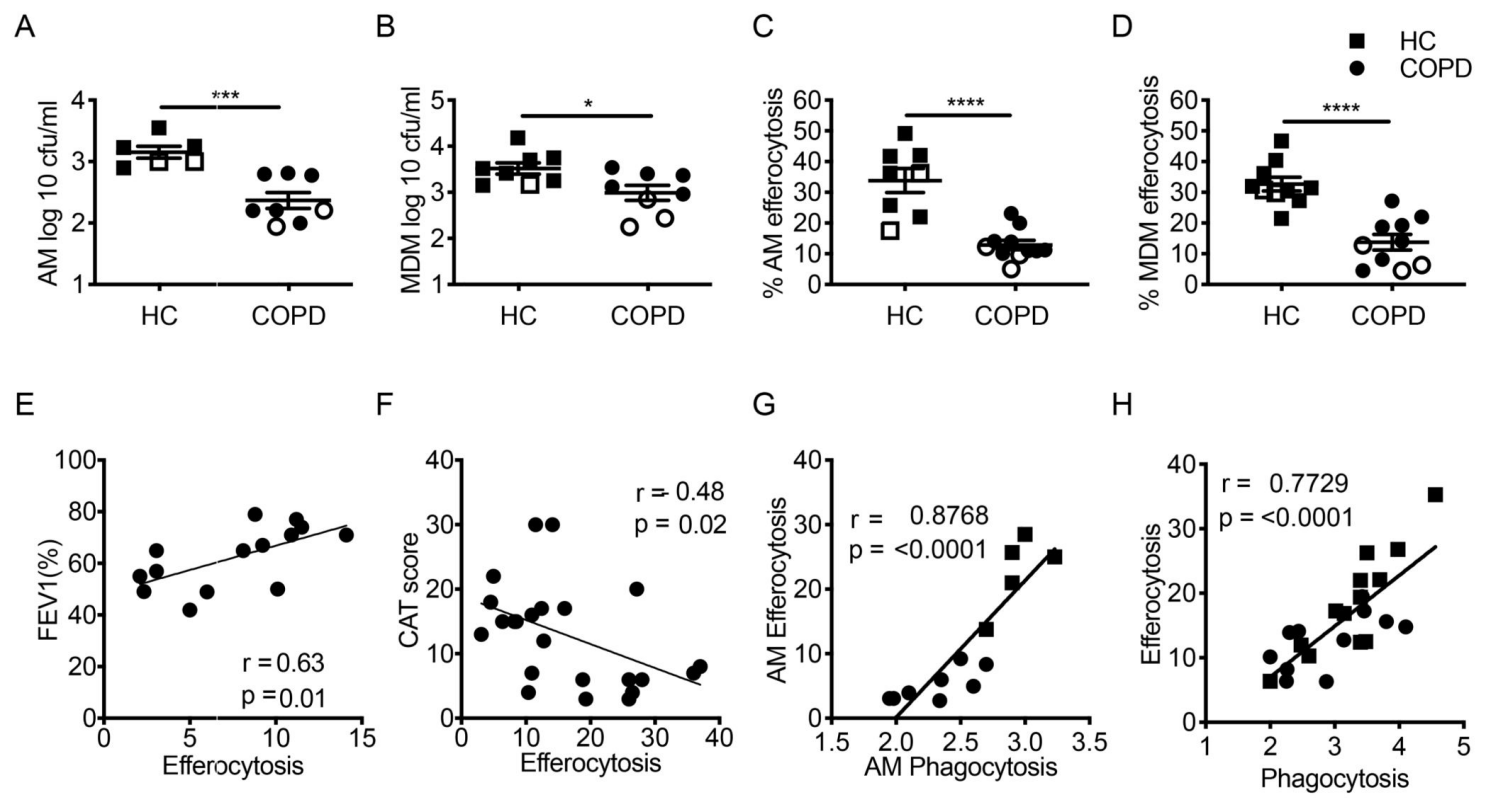
Intrinsic defects in COPD alveolar and peripheral blood monocyte-derived macrophages. **(A-B)** Alveolar (AM) and Monocyte-Derived Macrophages (MDM) from healthy controls (squares) and COPD donors (circles) were challenged with opsonised serotype 14 *S. pneumoniae* for 4 hours and numbers of viable bacteria measured or **(C-D)** co-incubated with PKH26 labelled 20h apoptotic neutrophils and efferocytosis rates measured by flow cytometry. (A, HC n=6, COPD n=8, B, HC/COPD n=8, C, HC n=8, COPD n=11; D, HC/COPD n=10). (**E-F**) COPD AM and MDM efferocytosis were correlated with FEV1% (E, n=14) and symptoms of disease severity, as measured by the CAT score (F, n=24). (**G**-**H**) Efferocytosis of PKH26 labelled apoptotic neutrophils was correlated with phagocytosis of *S. pneumoniae* S14 in both Alveolar (G, n=13) and Monocyte- Derived Macrophages (H, n= 24) from COPD and healthy donors. Open squares and circles represent current smokers. Data represent individual values with mean ± SEM. P values calculated by unpaired t-test, *P ≤0.05, ***P≤0.001, ****P≤0.0001. Pearson’s correlation coefficient (r) as shown.

Alveolar macrophages (AM) and peripheral monocyte-derived macrophages (MDM) were compared to interrogate intrinsic and extrinsic (niche dependent) factors impacting on macrophage function. Both AM and MDM isolated from patients with COPD demonstrated defective phagocytosis of live opsonised S14 *S.pneumoniae* (Fig. 1A-B), and efferocytosis of PKH26 labelled apoptotic neutrophils compared to healthy controls (HC) (Fig. 1C-D), as previously described^7,20^. Rates were lowest in current smokers across both processes, as expected^21^, but macrophage dysfunction did not recover in COPD patients who were no longer active smokers (Fig. 1A-D). To establish if there was a direct clinical read out of impaired efferocytosis in COPD, we performed correlation analysis of macrophage efferocytosis against FEV1. There was a significant relationship observed between macrophage efferocytosis and FEV_1_ % predicted (Actual Donor FEV1 as a % of their predicted FEV1, based on height and age) (Fig. 1E). Similarly, we observed a negative correlation between COPD macrophage efferocytosis and high CAT scores, representative of increased symptom severity (Fig. 1F). Correlation between bacterial phagocytosis in COPD macrophages and both FEV1 and high CAT scores has previously been published by our group^7^. Macrophage bacterial internalization (fig. E1A) and efferocytosis (fig. E1B) did not correlate with study participant age. Crucially, there was a high degree of correlation in both AM and MDM between bacterial phagocytosis and efferocytosis within each study participant (Fig. 1G-H). Together with conservation of phenotype between AM (Tissue) and MDM (blood) derived cells, these observations led us to question whether a shared intrinsic defect was driving impaired phagocytosis and efferocytosis in COPD macrophages.

### COPD macrophages demonstrate a baseline defect in metabolic processing

**Figure 2:**
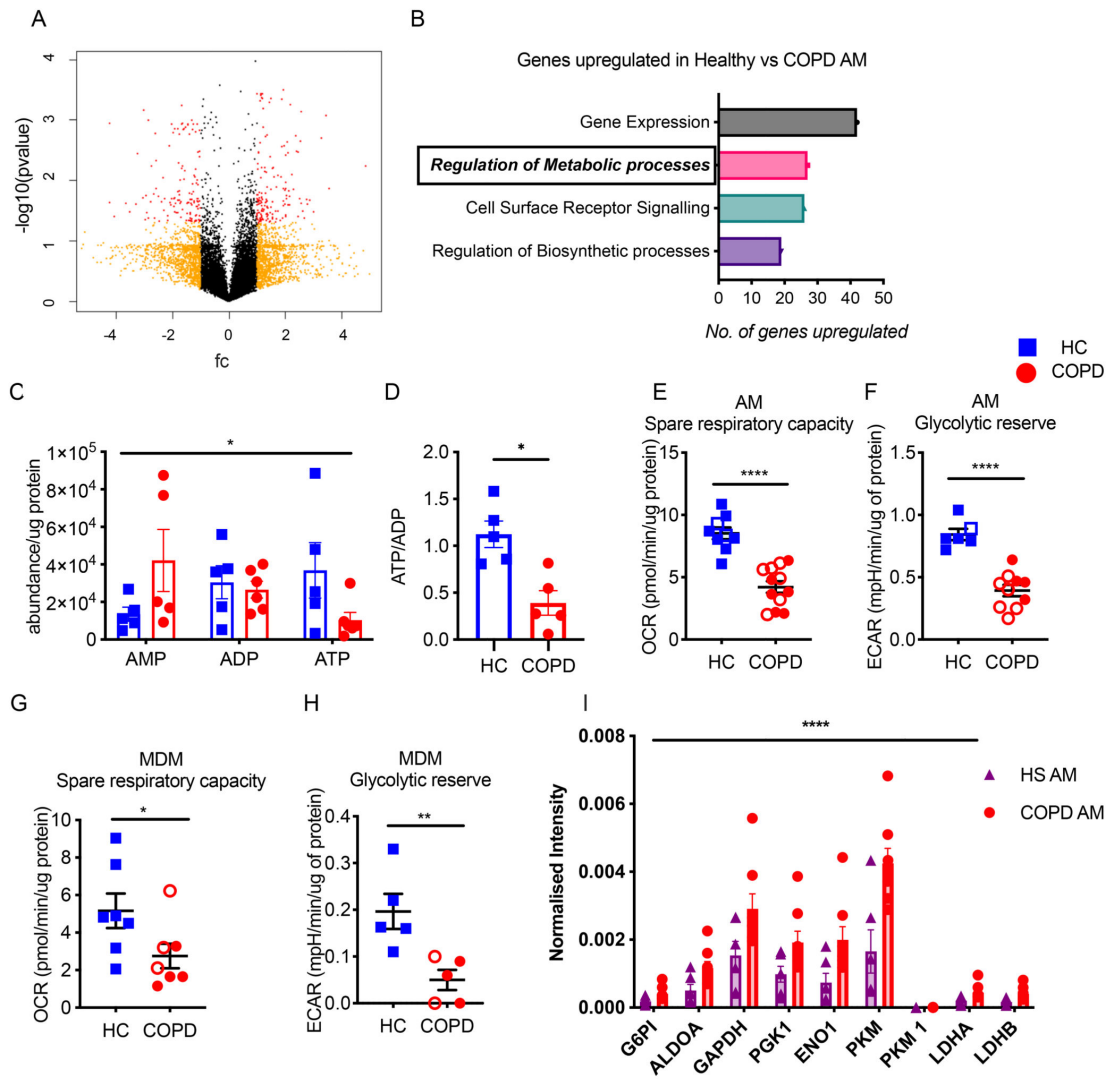
Loss of energy reserves in COPD macrophages. (**A**-**B**) AM were isolated from COPD and healthy donors via bronchoalveolar lavage, cultured for 16 hours prior to collecting RNA for Total RNA-seq, n=3. **(A)** Volcano plots displaying the log_2_ fold change (FC) between COPD and healthy donors. Red dots (n=287) represent genes which are significantly altered. Orange dots represent genes which are altered but do not meet significance. **(B)** Top four Gene Ontology (GO) processes upregulated in healthy AM, at baseline, compared to COPD AM. (**C**-**D**) HPLC-MS analysis was carried out to determine the levels of ATP, ADP and AMP in resting state healthy donor (squares) and COPD (circles) AM and energy charge ([ATP+1/2ADP]/ (ATP+ADP+AMP]) was calculated (n=5/6). (**E**-**H**) Seahorse mitochondrial **(E, G)** and glycolytic **(F, H)** stress testing in COPD AM and MDM. (E, HC n=9, COPD n=12, F, HC n=6 COPD n=10, G, n=7, H, n=5). Open squares and circles indicate current smokers. COPD Smoker vs Ex-Smoker, E, p value= 0.103, F, p value= 0.158, G, p value = 0.17. **(I)** Glycolytic enzyme abundance in COPD (n=7) versus “Healthy Smoker” (HS) AM (n=5) as determined by data-independent acquisition MS proteomic analysis. Data represents individual values ± SEM. (A-B) Significance determined at FC >log_2_1.5 and p value ≤ 0.05. P values calculated via (C,I) 2-way ANOVA, (E,F,G) unpaired t-test, (D+H) Mann Whitney U Test. *P ≤0.05, **P≤0.01, ****P≤0.0001. ECAR= Extracellular acidification rate, OCR= oxygen consumption rate. HPLC-MS= High performance liquid chromatography mass spectrometry. FC =Fold change.

To explore intrinsic differences in baseline transcript abundance in AM isolated from patients with COPD and healthy controls, we undertook Total RNA-Seq of AM isolated from bronchoalveolar lavage, as detailed in the online supplement. Transcriptional analysis revealed a total of 287 genes to be differentially expressed between resting state COPD and healthy AM (Fig. 2A). In keeping with previous studies in COPD patients, we found metabolic processes to be one of the most significantly suppressed in COPD AM (Fig. 2B)^22,23^. As reprogramming of macrophage metabolism, particularly mitochondrial metabolism^24^, is inextricably linked to function and activation states^25,26,27,28^, we questioned if altered metabolism in COPD macrophages results in defective cellular energetics and impaired effector function. To firstly define the energetic states of isolated AM, we undertook LC-MS analysis comparing COPD patient AM with healthy control AM. COPD AM revealed a significantly altered abundance of ATP (Fig. 2C) and concurrent reduction in energy charge status (ATP:ADP) (Fig. 2D). To establish if the altered energy state observed in COPD AM was consequent upon changes in oxidative or glycolytic metabolism, we used seahorse metabolic profiling to define basal metabolic states and metabolic reserve capacity, defined as the difference between basal and peak metabolic rates recorded after injection of metabolic stressor compounds, as outlined in the online supplement. COPD AM demonstrated equivalent extracellular acidification rates (ECAR) (fig. E2A) and reduced oxygen consumption rates (OCR) (fig. E2B) at baseline compared to healthy controls. In addition to the previously reported reduction in spare respiratory capacity^22^, we observe a loss of both oxidative (Fig. 2E) and glycolytic reserve capacity (Fig. 2F) in COPD AM following exposure to metabolic stressors. Despite equivalent basal respiration and glycolytic rates (fig. E2C-D), cellular energetics were also defective in peripherally derived MDM from COPD donors, with depleted reserves in both oxidative metabolism (Fig. 2G) and glycolysis (Fig. 2H). In COPD donors, both AM and MDM reserves were unaffected by smoking status, suggesting changes in metabolism are hardwired. Moreover, changes in glycolytic enzyme abundance observed in COPD vs “healthy smoking” control AM, as detected by data-independent acquisition MS proteomic analysis, would suggest that skewing towards glycolysis is a disease specific response and not a consequence of smoking status (Fig. 2I). While Healthy AM increased their maximal respiration rate following co-incubation with apoptotic neutrophils (AN), COPD AM, in contrast, failed to exhibit any uplift in energetic capacity (fig. E2E and E2EF). Treatment of healthy donor AM with established M2 polarisation stimuli^28,29^ did not induce a change in respiratory reserve, suggesting the spare respiratory capacity observed in healthy AM is a fundamental feature of these cells rather than a polarisation state (fig. E2G). Additionally, we did not observe a predominance of M1 or M2 markers of those detected in the proteome of COPD versus Healthy donor AM^30,31^ (fig. E2H).

### Defects in oxidative metabolism drive metabolic exhaustion in COPD macrophages

**Figure 3:**
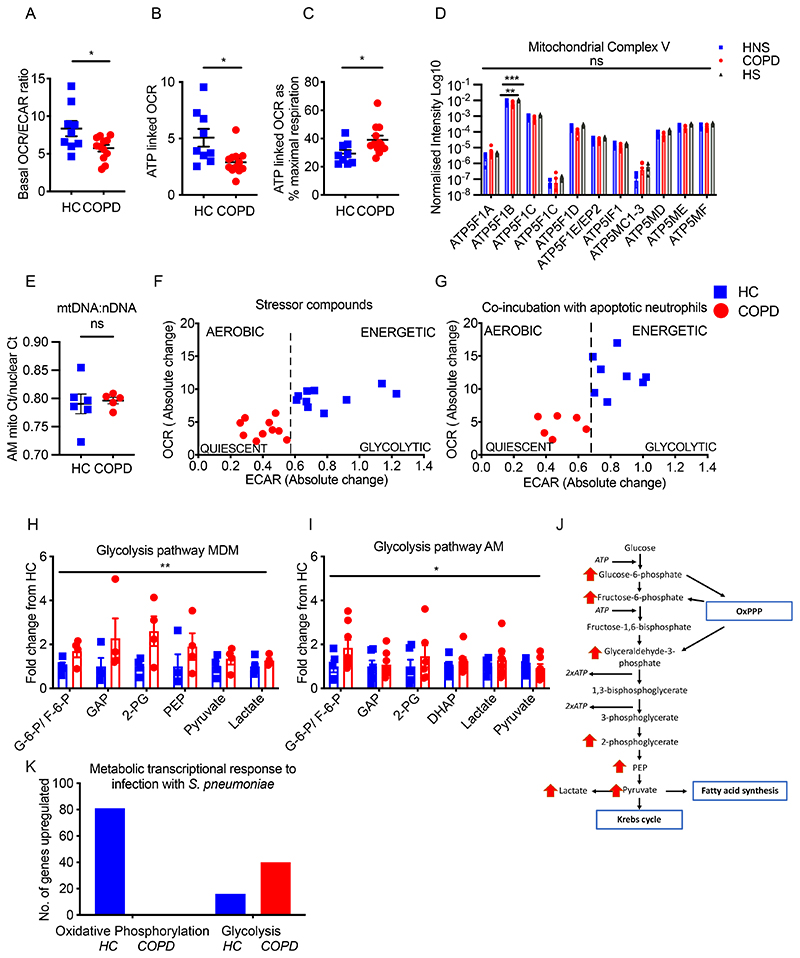
COPD macrophages display a preponderance for glycolytic metabolism. (**A**) Calculated OCR/ECAR ratio in healthy (squares) and COPD (circles) AM (**B**) OCR consumption linked to ATP generation in healthy and COPD AM as calculated by the reduction in OCR following oligomycin treatment. (**C**) ATP-linked-OCR as a percentage of maximal OCR in healthy and COPD AM. HC n=9, COPD N=12. **(D)** Relative protein abundance of the 11 detected subunits of Mitochondrial ATP Synthase/ Complex V, as measured by data-independent acquisition MS proteomic analysis. COPD AM n= 7, Healthy Non smoker (HNS) and Healthy Smoker (HS) n= 5. (**E)** AM Mitochondrial DNA to Nuclear DNA ratios, as measured by Real-Time quantitative PCR. Healthy donor n= 6, COPD donor n= 5. (**F**-**G**) Absolute change in ECAR was plotted against absolute change in OCR following injection of mitochondrial stressor compounds in resting macrophages (F, n=21) and after cells were co-incubated with 20h apoptotic neutrophils for 90 min (G, n=14). (**H- I**) Glycolytic metabolite abundance determined by HPLC-MS in resting state MDM (H, n=4) COPD AM (I, HC n= 6, COPD n=8), plotted as relative to healthy control. **(J)** Schematic of glycolytic intermediaries increased throughout the glycolytic pathway in COPD macrophages. (**K**) The transcriptional response in COPD and healthy control AM following co-incubation with opsonised D39 *Streptococcus pneumoniae*, n=3. Data represents individual values and mean ± SEM. P values calculated by (A-C) unpaired t-test, (E) Mann Whitney U Test, (D,H,I) via 2-way ANOVA. *P≤0.05, **P≤0.01, ns= not significant, FC= Fold Change.

With reductions in both oxidative and glycolytic reserve observed, we next questioned which metabolic processes predominate at baseline and in challenged states. Compared to healthy AM, COPD AM had a significantly reduced OCR/ECAR ratio (Fig. 3A). This preponderance for glycolysis over oxidative metabolism was again demonstrated by significantly reduced ATP-linked-OCR in COPD AM compared to healthy AM (Fig. 3B). Interestingly, while absolute ATP- linked OCR was reduced, it did represent a significantly higher percentage of maximal respiration in COPD AM during mitochondrial stress testing (Fig 3C). This suggests a lack of redundancy in mitochondrial units in COPD AM, whereby these cells are seemingly unable to recruit additional mitochondria or to further increase ATP production in existing mitochondria. Strikingly, this occurred in the presence of comparable protein abundance of the critical ATP generating complex, Mitochondrial ATP Synthase or Complex V subunits (Fig. 3D), conserved mitochondrial to nuclear DNA ratios and comparable abundance of key mitochondrial fusion and fission proteins (Fig. 3E, fig. E3A).

To evaluate how cells differentially employed either metabolic pathway to meet increased energy demand, absolute change in ECAR was plotted against absolute change in OCR, following injection of stressor compounds. While healthy AM increased both ECAR and OCR rates, COPD AM revealed a defective induction in oxygen consumption rates (Fig 3F), a response magnified when concurrently challenged to undergo normal effector function by efferocytosing apoptotic neutrophils (Fig. 3G). Thus, COPD macrophages fail to induce oxidative metabolism with an over reliance on glycolytic energy.

To more directly identify metabolic blocks in COPD, we undertook HPLC-MS analysis of both COPD and healthy donor macrophages at rest. COPD MDM (Fig. 3H) and AM (Fig. 3I) both demonstrated a significant increase in abundance of glycolytic intermediaries compared to healthy control cells, supporting a glycolytic switch in the context of COPD (Fig. 3J). This was independent of BAL nutrient availability (fig. E3B-C), macrophage glucose uptake (fig. E3E-G) or glycogen storage (fig. E3H), suggesting differential substrate availability is not driving this phenomenon. Interestingly, BAL lactate, however, was significantly higher in COPD donors (fig. E3D). To investigate if the basal reliance on glycolysis and defective induction in oxygen consumption under conditions of activation and stress were regulated at a transcriptional level, we conducted a targeted analysis of a previously published affymetrix microarray data set from our group^7^,in which AM were cultured in the presence of *S. pneumoniae*, as detailed in the online supplement. In keeping with a failure to induce oxidative metabolism, COPD AM failed to induce any genes associated with oxidative phosphorylation in contrast to healthy control AM following exposure to *S. pneumoniae* (Fig. 3K). Conversely, upregulation of glycolytic genes in COPD AM represented 10% of the total genes upregulated in response to infection.

### Loss of malic enzyme 1, a critical regulator of macrophage oxidative metabolism and redox buffering, recapitulates COPD macrophage dysfunction

**Figure 4:**
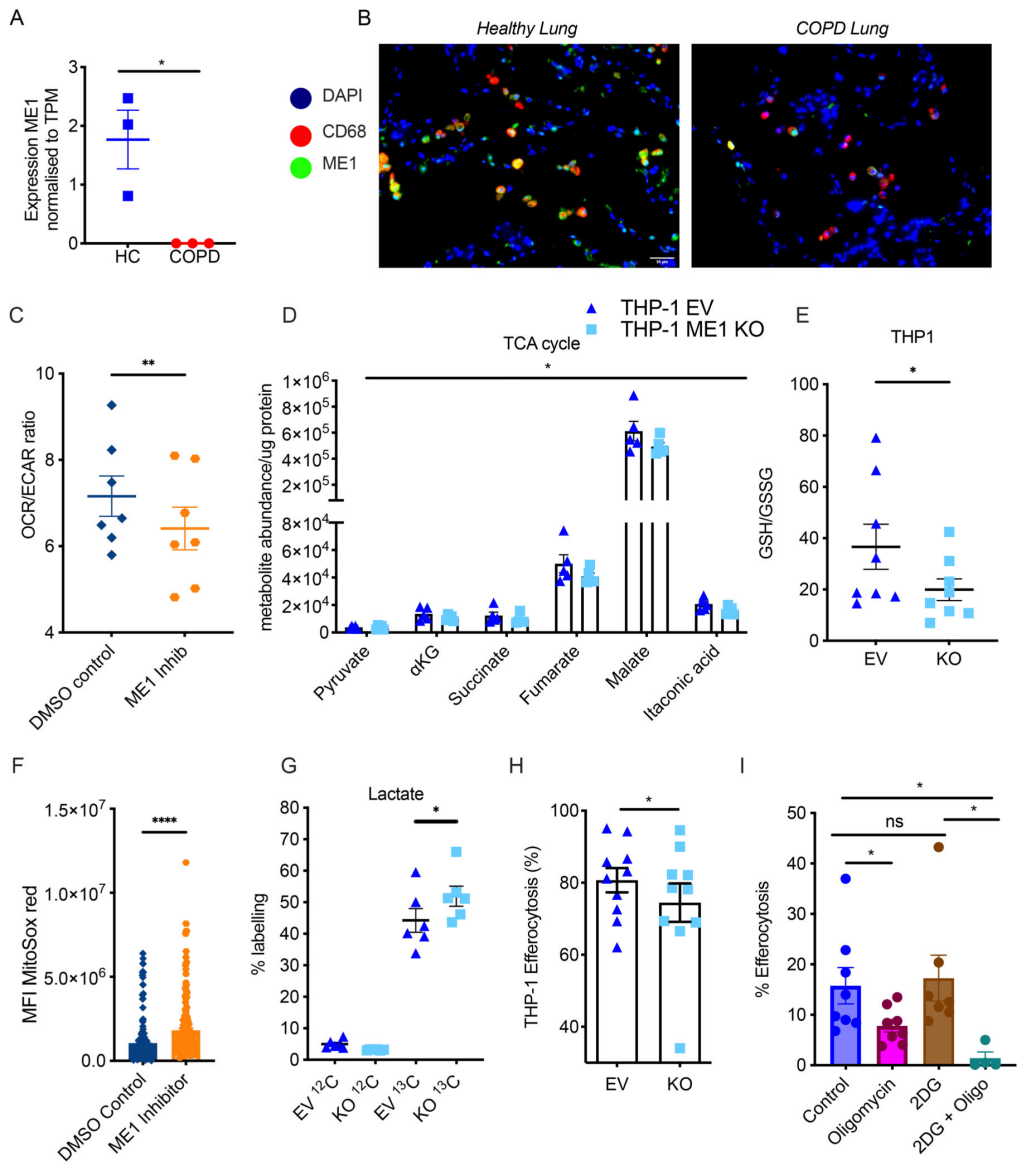
Malic enzyme 1 (ME1) plays a critical role in macrophage metabolism and efferocytosis. **(A)** Transcriptomic analysis of baseline *ME1* expression in healthy control and COPD AM normalised to TPM (transcripts per kilobase million), n=3 (**B**) ME1 expression in healthy and COPD patient lung sections prepared from paraffin-embeded blocks, images taken at x20 magnification (**C**) Healthy MDM were treated x16 hours with a chemical ME1 inhibitor or DMSO control before measuring the basal OCR:ECAR ratio on a Seahorse platform, n=7. (**D**) HPLC-MS analysis of TCA cycle metabolite abundance in THP-1 empty vector control (EV) and *ME1* knockout (*ME1* KO) cells, normalised to protein content, n=5. **(E)** GSH:GSSG ratio calculated in THP-1 EV and *ME1* KO cells, n=9. **(F)** Healthy MDM were treated x16 hours with a chemical ME1 inhibitor or DMSO control. Mean fluorescence intensity of MitoSox Red was then measured from >180 cells per condition for n= 3 donors. **(G)** U-^13^C glucose incorporation into lactate in THP-1 EV and *ME1* KO following 6h of culture in U-^13^C glucose containing media, n=6. **(H-I)** THP-1 EV and *ME1* knockout cells (H) and healthy MDM pretreated with Oligomycin 2μM, 2DG 50mM or Oligomycin and 2DG combined for 1hr (I) prior to co-incubation with PKH26 labelled 20h apoptotic neutrophils for 90 minutes and efferocytosis rate determined by flow cytometry, (H) n=8 (I) n= 8,8,7 and 4 respectively. Data represents individual values and mean ± SEM. P values calculated via (A) adjusted t-test values from Total RNA-seq analysis as outlined in online supplement, (C,E,G,H) paired t-test, (D,I) 2-way ANOVA with Dunnett’s multiple comparisons, (F) Wilcoxon matched paired signs rank test. *P≤0.05, **P≤0.01, ****P≤0.0001, ns= not significant. HPLC-MS = High performance liquid chromatography mass spectrometry.

Further analysis of the metabolic signature detected by Total RNA-Sequencing of COPD versus Healthy donor AM (Fig. 2A) revealed a complete downregulation of malic enzyme 1 transcript (*ME1*) in COPD AM (Fig. 4A). This was associated with a marked reduction in ME1 protein expression within the lung macrophage compartment in patients with COPD (Fig. 4B). ME1 catalyses the reversible oxidative decarboxylation of malate to pyruvate, resulting in replenishment of TCA cycle intermediaries and the conversion of NADP+ into NADPH. As a consequence loss of ME1 has been shown to disrupt redox balance and antioxidant defence with reduced GSH:GSSG ratios^32^ and induction of HO-1 in PC3 and HCT116 cell lines^33^. Thus, we sought to assess whether suppression of macrophage *ME1* recapitulates the metabolic defects observed in COPD. We used a CRISPR cas9 system to delete *ME1* from the THP-1 macrophage cell line (fig. E4A) coupled with a chemical ME1 inhibitor in healthy MDM cells. In keeping with our observed COPD phenotype, *ME1* loss resulted in a reduction in OCR:ECAR ratios (Fig. 4C) and a reduction in TCA cycle intermediaries (Fig. 4D). These metabolic adaptations were associated with a reduction in redox capacity as evidenced by a reduced GSH:GSSG ratio and high basal mROS levels (Fig. 4E-F). A proportional increase of ^13^C glucose incorporation into lactate in *ME1* deficient cells (Fig. 4G), as previously reported in the HCT116 cell line^33^, revealed that in the setting of impaired oxidative phosphorylation, macrophages adapted by increasing glycolytic flux. Importantly, this observed increase in glycolysis was, however, insufficient to confer effective macrophage function, as evidenced by reduced efferocytosis rates in *ME1* KO cells, mirroring the defects detected in COPD macrophages (Fig. 4H). In keeping with a critical role for oxidative phosphorylation in macrophage efferocytosis, healthy MDM treated with oligomycin also displayed suppressed efferocytosis (Fig. 4I). Glycolysis only contributed to efferocytic capacity when oxidative phosphorylation was blocked (Fig. 4I).

As ME1 loss has been associated with senescence^33^ and senescence has been linked with skewing towards glycolysis in human fibroblasts^34^, we next surveyed established markers of senescence^35^, in the proteome of AM recovered from healthy non-smokers, “healthy smokers” (defined as current smokers with normal spirometry) and patients with COPD. There was no consistent pattern of expression to support a senescent switch within the COPD AM accounting for the change in metabolic and effector function (fig. E5A-F). We therefore propose that ME1 plays a key role in dictating redox buffering capacity and metabolic flux in macrophages and that loss of ME1 skews cells towards glycolysis and leads to impaired effector function. Metabolic and functional phenotyping of ME1 loss recapitulated core elements of COPD macrophage dysfunction, establishing ME1 to be an important regulator of macrophage function in both health and disease states.

### Activation of NRF2 rescues ME1 expression, reprogrammes metabolism, improving cellular energetics and redox balance and restores function in COPD Macrophages

Previous GWAS^36^, cellular^13,37,38^ and murine^39^ studies have identified a potential role for the NRF2 mediated anti-oxidant transcriptional response in the pathogenesis of COPD. More recently work from our group has shown that augmentation of NRF2 activity can improve bacterial phagocytic capacity in COPD macrophages^7^. Whilst the mechanisms by which NRF2 agonists recover COPD macrophage effector function remain to be fully elucidated, previous studies have suggested that NRF2 augmentation of non-opsonic bacterial phagocytosis in COPD occurs in part via upregulation of the scavenger receptor MARCO^13^. We observe loss of expression of both MARCO and the NRF2 independent Class A scavenger receptors SRA1 and SRA4 in COPD AM (fig. E6A-C). In MEFS isolated from mice lacking *Nrf2*, *Nrf2* has also been shown to modulate mitochondrial respiration and ATP levels ^40^. Moreover, studies in human lymphoid and cancer cell lines have identified ME1 to be a target gene of NRF2^41,42^. Given the inherent shared defect we observed in both efferocytosis and opsonic phagocytosis, we therefore questioned whether a specific NRF2 agonist KI-696^7,43^, could in part mediate its effect by overcoming the intrinsic metabolic defects observed in COPD AM and MDM.

**Figure 5:**
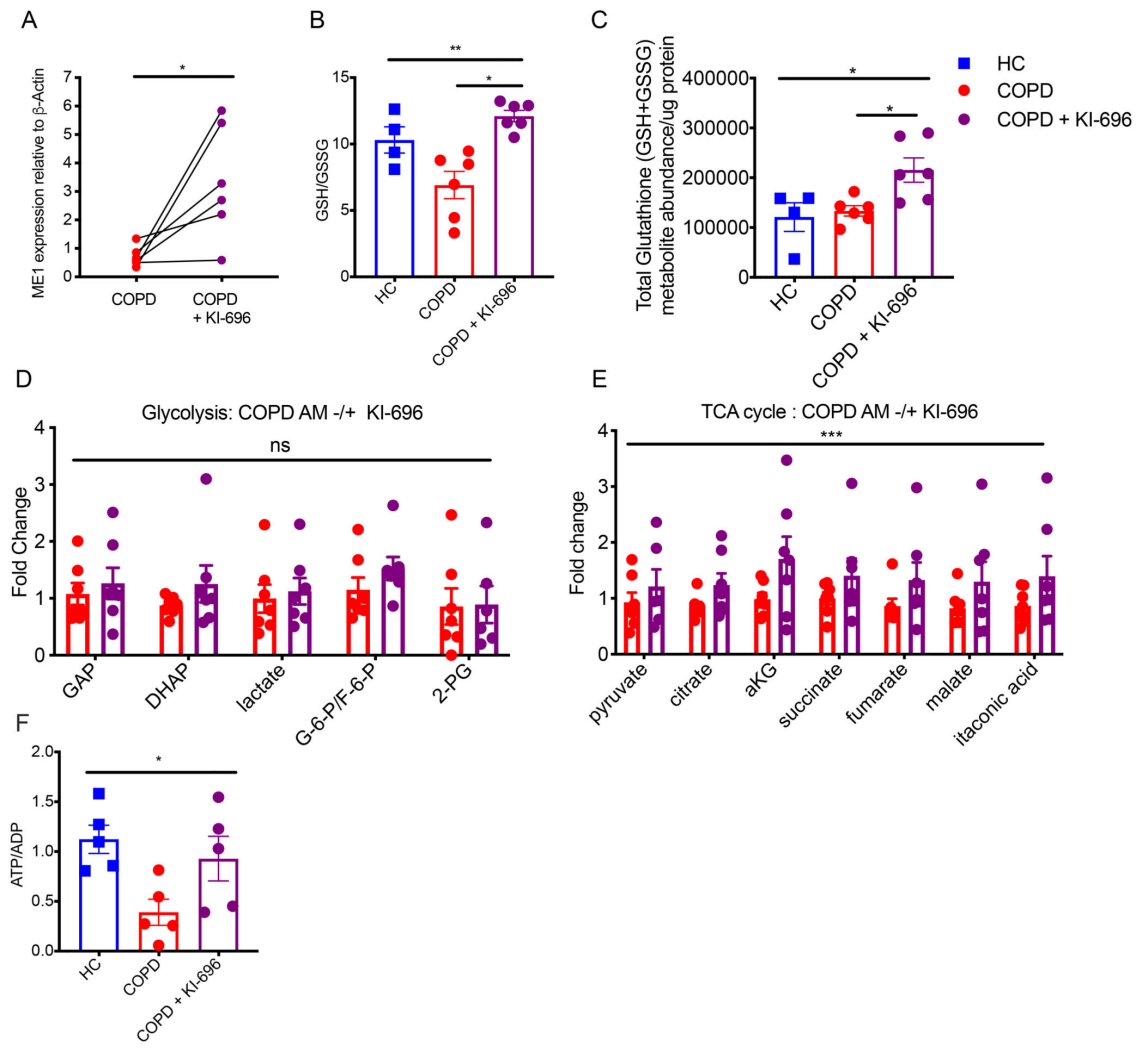
Cellular energetics and redox status are improved in COPD AM following activation of ME1 via the NRF2 agonist KI-696. (**A**) Real-time PCR quantification of *ME1* expression relative to β-actin in COPD AM following culture in the absence or presence of KI-696, n=6. **(B-C)** Treatment with Ki-696 alters redox protection in COPD AM as measured by (B) GSH:GSSG ratios and (C) absolute total glutathione abundance (GSH and GSSG), HC n= 4, COPD n=6. (**D-E**) LC-MS analysis of healthy and COPD AM (+/- KI-696). Metabolite abundance is plotted as relative to untreated COPD AM (fold change) n= 7. **(F)** Energy status expressed as an ATP to ADP ratio was calculated, n=5. Data represents individual values and mean ± SEM. P values calculated via (A) paired t-test, (B,C,F) Kruskal Wallis with Dunn’s multiple comparison tests and (D-E) 2-way ANOVA. *P≤0.05, **P≤0.01, ***P≤0.001.

Activation of NRF2 via KI-696 significantly increased *ME1* expression in COPD macrophages, as anticipated (Fig. 5A). In keeping with the established role for ME1 in generating NADPH, we also observed an improvement in GSH:GSSG ratios and an increase in total glutathione availability in COPD macrophages following treatment with KI-696 (Fig. 5B-C). To more directly measure the effects of NRF2 agonists on the metabolic capacity of COPD AM, we undertook LC-MS analysis of AM from COPD patients treated with KI-696 and compared these to untreated COPD and healthy control AM. KI-696 mediated NRF2 activation did not significantly alter glycolytic metabolite abundance (Fig. 5D). However, it did significantly increase the abundance of all TCA cycle intermediaries relative to baseline abundance in untreated COPD AM (Fig. 5E) and partially restored cellular energetics (Fig. 5F).

**Figure 6:**
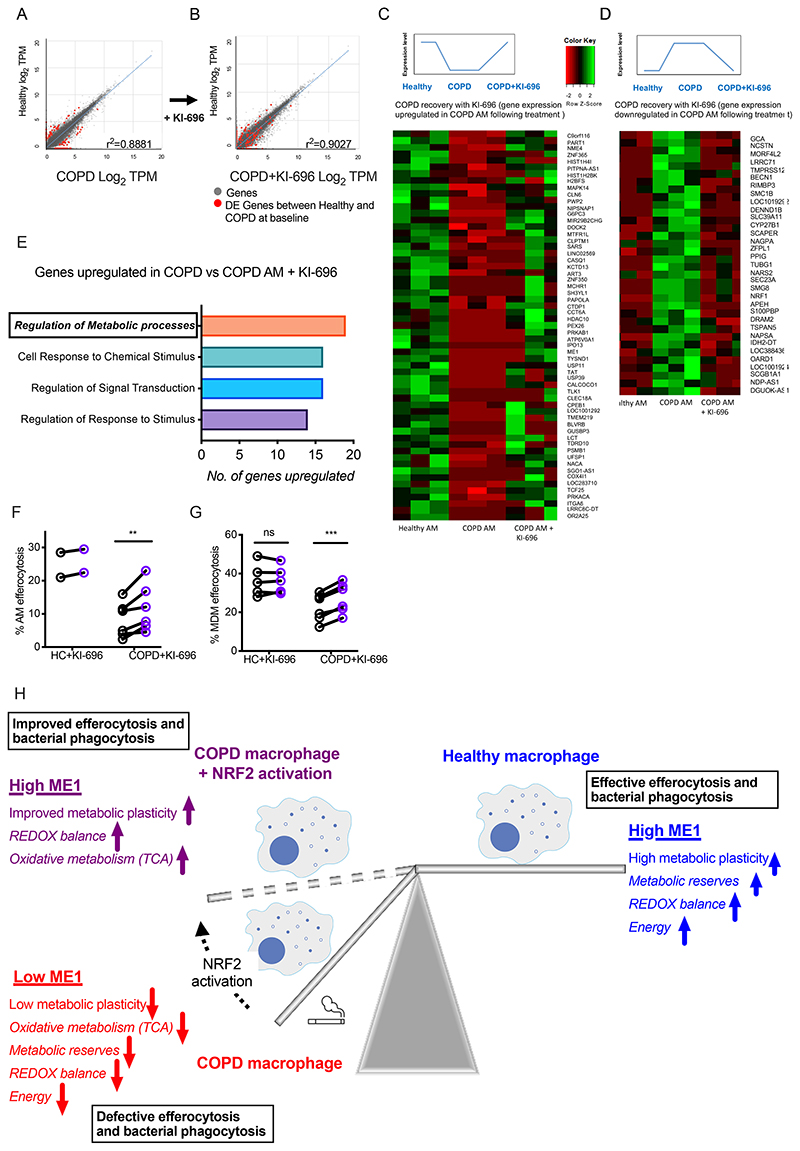
Activation of NRF2 with KI-696 reprogrammes alveolar macrophages in COPD with consequences for effector function. (**A**-**E**) Healthy and COPD AM were cultured for 16h (+/- KI-696) prior to collection of RNA for Total RNA-seq, n=3. **(A-B)** Correlation analysis between healthy and COPD AM pre and post treatment with KI-696. Red dots represent significantly differentially expressed genes between COPD and Healthy AM at baseline, which are seen to move towards the trendline in (B). **(C-D)** Heatmap of normalized Z-scores showing genes which were initially comparatively downregulated (C) or upregulated (D) in COPD vs healthy AM, which were transcriptionally reprogrammed by treatment with KI-696, in the direction of healthy AM. (**E**)The lead Gene Ontology (GO) terms upregulated in COPD AM following treatment with KI-696. **(F**-**G**) COPD AM (F, HC n=2, COPD n=6) and MDM (G, HC n=5, COPD n=6) were pre-treated for 16h with the NRF2 activator KI-696, prior to co-incubation with PKH26 labelled 20h apoptotic neutrophils and measurement of efferocytosis rates by flow cytometry.(H) Summary diagram of metabolic changes in COPD macrophages and the role of NRF2 augmentation. Data represents individual values and mean ± SEM. (A-B) Scatter plots were generated by plotting the average Log_2_ Tags Per Million (TPM) scores for healthy AM vs the average Log2 TPM scores for COPD AM +/- KI-696. R^2^ squared values were calculated from the slope of the correlation trendline. DE genes= FC > log_2_1.5 and P value≤0.05. (F-G) P values calculated via (F) paired t-test (G) COPD donors paired t- test and healthy donors Wilcoxon matched paired signs rank test. **P≤0.01, ***P≤0.001.

To address the processes underlying this metabolic rescue, RNAseq analysis was performed on AM from healthy donors and COPD patients at baseline and following treatment with KI-696. Correlation analysis across the 30,000 gene set revealed that COPD AM were more highly correlated with healthy AM following treatment with KI-696 (Fig. 6A-D). “Regulation of metabolic processes” was the most differentially upregulated biological process in COPD AM following NRF2 activation (Fig. 6E). Amongst *ME1*, other metabolic genes of interest altered by NRF2 activation of COPD AM, included G6PC3, MAPK14, TLK1 and OARD1 (Fig 6C-D). Finally, we questioned if the NRF2 mediated restoration of redox balance and correction of disturbances in oxidative metabolism and cellular energetics could rescue COPD macrophage efferocytosis. COPD MDM and AM both improved efferocytic capacity in the continued presence of the NRF2 agonist KI-696 (Fig 6F-G and fig. E6D). Failure of KI-696 to recover efferocytosis in *ME1* KO cells, would suggest that augmentation of ME1 expression is required for NRF2 mediated enhancement of efferocytosis (fig. E6E). Figure 6H summarizes the functional outcomes associated with impaired metabolic plasticity in COPD macrophages and therapeutic rescue with NRF2 activation.

## Discussion

COPD is characterized by persistent inflammation of the airways, destruction of lung tissue and mucus hypersecretion resulting in airflow limitation and impaired gas exchange. Pathogen colonization and recurrent infective exacerbations are the largest contributor to morbidity and mortality within the disease^3^. Failure of COPD macrophages to adequately phagocytose and kill bacteria and to instigate inflammation resolution via efferocytosis, places macrophage dysfunction at the centre of both disease pathology and progression in COPD^5,12,44^. Study of alveolar macrophages, directly influenced by the lung microenvironment and peripherally derived monocyte-derived macrophages, has enabled us to define an intrinsic metabolic defect in COPD macrophages which drives this functional impairment. Extending from previous descriptions of diminished baseline respiratory reserve capacity in COPD AM^22^, we describe a more global defect in COPD macrophage bioenergetics when these cells are engaged to undertake the highly energy requiring process of efferocytosis. Moreover, we provide evidence that defective metabolism is not an exclusive consequence of an inflamed pulmonary niche by also characterising this metabolic exhaustion, although to a lesser degree, in peripherally circulating MDM. Within the limitations of the small number of healthy smoker controls included in this study, defective metabolism would also appear to be independent of current smoking status, with evidence of alterations in glycolytic enzyme abundance specific to disease state. This leads us to postulate that there is both central (bone marrow) reprogramming of newly formed monocytes which contribute to replenishment of the alveolar macrophage compartment in the diseased lung and peripheral (lung tissue) reprogramming of the alveolar macrophage compartment following exposure to the local inflammatory milieu and metabolic intermediaries. This concept is supported by emerging evidence that reprogramming of myelopoiesis has consequence for myeloid cell tissue effector functions in acute^45^ and chronic inflammatory disease states^6,24^, with overlapping transcriptional signatures previously reported in COPD MDM and AM populations^46^ and evidence of changes in DNA accessibility and methylation marks in the AM compartment during trained immune responses^47,48^.Metabolic plasticity is a crucial feature of macrophage adaptability, with the capacity to generate ATP via multiple metabolic pathways, namely glycolysis, oxidative phosphorylation and fatty acid oxidation^25,28^. Recent evidence has demonstrated that the dichotomy of glycolysis supporting acute inflammatory responses and oxidative metabolism fuelling sustained energy production is as an over simplification of macrophage bioenergetics, with significant cross talk required between these metabolic pathways^49,50,51^. Importantly, we describe a refractory metabolism in COPD macrophages with both a significant depletion in glycolytic and mitochondrial respiration derived energy reserves and an over reliance on glycolysis as a source for ATP. Further work will be required to understand how these intrinsic metabolic adaptions influence and are themselves influenced by changes in the wider pulmonary niche as exemplified by elevated BAL lactate levels in COPD donors. This vulnerability of COPD macrophages to defects in reserve energy capacity has consequence for key effector functions, with glycolysis only partially supporting healthy macrophage efferocytosis in the absence of oxidative phosphorylation.

With high oxidative stress, mitochondrial dysfunction and suppressed oxidative phosphorylation features of COPD ^14,23,52,53,54^, we were interested to note the basal suppression of transcript for the enzyme, malic enzyme 1 (ME1), in COPD macrophages. ME1 is the rate regulating enzyme for the cytosolic malate-pyruvate shunt. In catalysing malate conversion to pyruvate, it generates NADPH, augmenting the replenishment of reduced glutathione for redox power^32^ and in turn shuttling pyruvate into the TCA cycle^55^. Replicating ME1 loss in vitro reveals its critical role in macrophage redox balance, with resulting changes in basal mROS levels, but also highlights that loss of ME1 skews macrophage metabolism away from oxidative metabolism, with consequence for effector function such as efferocytosis. The specific mechanisms by which ME1 loss drives increased glycolytic flux remain to be fully explored, and may relate not only to redox capacity but also a need to replenish intracellular pyruvate levels. To date, there is no evidence linking ME1 activity directly to macrophage polarisation states. Specifically, a malic enzyme signature was not detected in the seminal paper on macrophage phenotype metabolism by Jha et al^28^. The specific mechanisms driving the depletion of ME1 in COPD are a subject for future work. Of note however, we observe changes in ME1 expression to occur at both a protein and transcript level. Coupled with evidence of altered activity of the ME1 transcriptional regulator NRF2, in the small airways of patients with COPD^56^, global changes in DNA methylation states, and correlation between HDAC2 and NRF2 expression in COPD MDM^57^, this supports the concept of intrinsic rewiring of macrophage responses. We speculate that epigenetic changes within the bone-marrow compartment would enable this reprogramming of the mononuclear phagocyte system with further modification by local cues within the airways of patients with COPD. We propose that it is the interplay between these central and peripheral epigenetic programs that regulates ME1 expression, with consequent changes in AM and MDM core effector functions.

Whilst ME1 was the only gene directly regulated by NRF2 which showed differential regulation at baseline in RNA-seq data sets comparing healthy control AM with COPD AM, previous work by our group has demonstrated a relative increase in protein expression of the NRF2 targets GLCL, NQO1 and HO-1 following NRF2 activation of COPD macrophages^7^. It is likely, therefore, that ME1 is one of a number of factors contributing to the metabolic defects observed in COPD and responsive to NRF2 augmentation. This is further supported by our observation that other metabolic genes including G6PC3, MAPK14,TLK1 and OARD1 are altered by activation of NRF2 activation in COPD AM, and the incomplete rescue of COPD macrophage effector function by KI-696. Understanding how regulators of transcriptional programmes and metabolic plasticity in COPD macrophages interact with processes that are responsive to NRF2 augmentation to rescue effector function will be essential for the development of new therapeutic strategies. Equally, study of patients with early disease and smokers who have airway inflammation but are yet to develop airflow obstruction, will also be of importance in defining the temporal relationship between defective macrophage metabolism and disease progression.

## Supplementary Material

Online Data Supplement

## Figures and Tables

**Table 1:** Demographics of study participants. Healthy Non-smokers = Lifelong non-smokers with normal spirometry. Healthy smokers = current smokers with normal spirometry. COPD donors = current or ex-smokers with an FEV1/FVC ratio of <0.70.

	Healthy Non smokers	“Healthy Smokers”	Subjects with COPD
No of Subjects	24	9	52
Age (yr)	S4 (31-70)	53 (36-68)	61 (39-77)
Sex female/male	12/12	2/7	29/23
FEV1 (L)	3.1 ± 0.7	3.5 ± 0.3	1.7 ± 0.5
FEV1 (% predicted)	105 ± 12.9	98 ± 4.1	64 ± 10.11
Gold Stage	N/A	N/A	14 Stage 1 26 Stage 2 11 Stage 3 1 Stage 4
Exacerbation per year 0/ >l/>2 / ≥3	N/A	N/A	14/10/20/8
Smoking status Current/Ex/Never	0/0/24	9/0/0	23/29/0
Pack years	n/a	30 ± 10	37 ± 14
CAT score (Max 40)	N/A	N/A	16 ± 8
Inhaled Medication: ICS+LABA / LAMA / SABA	N/A	N/A	25/27/34
