# Supplementary material for: NRF2 activation reprogrammes defects in oxidative metabolism to restore macrophage function in COPD
Source: Am J Respir Crit Care Med. Author manuscript; Available in PMC 2023 Apr 15. (PMC7614437; doi:10.1164/rccm.202203-0482OC)
Supplement: Online Data Supplement [file EMS164622-supplement-Online_Data_Supplement.pdf]

Online Data Supplement:

NRF2 activation reprogrammes defects in oxidative metabolism to restore macrophage function in COPD

Authors: Eilise M. Ryan[1], Pranvera Sadiku[1], Patricia Coelho[1,] Emily R. Watts[1], Ailiang Zhang[1], Andrew J.M. Howden[2], Manuel A. Sanchez-Garcia[1], Martin Bewley[3], Joby Cole[3], Brian J. McHugh[1], Wesley Vermaelen[4], Bart Ghesquiere[4], Peter Carmeliet[5,6,7], Giovanny Rodriguez Blanco[8], Alex Von Kriegsheim[8], Yolanda Sanchez[9], William Rumsey[9], James F. Callahan[9], George Cooper[1], Nicholas Parkinson[10], Kenneth Baillie[10], Doreen A. Cantrell[2] , John McCafferty[11], Gourab Choudhury[11], Dave Singh[12], David H. Dockrell[1], Moira K.B. Whyte[1*]†, Sarah R. Walmsley[1*]†.

Supplementary materials and methods:

Study Approval: Written informed consent was obtained and patients were recruited for bronchoscopy and venesection in accordance with local ethics (South East Scotland Research Committee , REC Ref 15/SS/0095 Greater Manchester South Rec ref 06/Q1403/156). Written informed consent was obtained from healthy volunteers for blood donation via the CIR Blood Resource Management Committee (AMREC 15-HV-013) in Edinburgh, United Kingdom.

Patient recruitment: Patients were recruited for bronchoscopy and venesection in accordance with local ethics. COPD donors had an FEV_1/FVC ratio of <0.70 , GOLD Stage 1,2,3 or 4 disease and were current or ex-smokers. Health status was evaluated via the validated COPD Assessment Test (E1). Healthy Bronchoscopy Donors (including "healthy smokers") had normal spirometry and were never, ex or current smokers. Patient demographics are outlined in Table 1. Patients were exacerbation free for at least 8 weeks and patients with diabetes, renal failure, liver failure, cardiac failure, active malignancy or other major respiratory diagnosis e.g. asthma or bronchiectasis were excluded. Patient samples were not randomized, but investigators were blinded to experimental conditions. Healthy volunteers for blood donation were age matched +/- 8 years .

Bronchoscopy: Bronchoalveolar Fluid (BAL) was collected from COPD and Healthy donors, as per ethics protocol. After inspection of the airways during bronchoscopy, the scope was wedged into the Right Middle Lobe (RML). 2-4 ml of lidocaine was then instilled via the scope into the RML, prior to injecting a total of 240ml of saline in 40ml

aliquots. The first 40ml of return was discarded/sent for routine culture and sensitivity, due to the high content of epithelial cells.

Seahorse Assays: After removal from standard culture plates, cells were seeded directly in to Xe24 seahorse assay plates (Agilent) at a density of 250,000 in 300 μl of monocyte-derived macrophages (MDM) and 210,000 in 300 μl per well of alveolar macrophages (AM) for 45 min at room temperature, before an additional 200 μl of complete medium was added to each well. All cells were subjected to either/both a glycolytic and mitochondrial stress test depending on cell numbers. For both Mitochondrial and Glycolytic stress test, 30ml of medium was made and pH adjusted as per the manufacturers guidelines (Agilent). Standard culture medium was replaced with 500μl of the appropriate Seahorse Stress Test Medium before incubating the plate at 37°C, with no CO_2 for 45 min. Glucose, Oligomycin , 2DG , FCCP and Antimycin & Rotenone were prepared as detailed below. Following the injection of each compound, three readings were taken 8 min apart totalling 3 cycles x 24 min each. Basal Glycolysis, Maximal Glycolytic Rate and Glycolytic Reserve (Glycolytic Stress Test) or Basal Oxygen Consumption, Maximal Respiratory Capacity, Spare Respiratory Capacity and Proton Leak (Mitochondrial Stress Test) were calculated during the assay and exported to Excel for analysis via Wave software (Agilent). ECAR & OCR readings were then normalised to μg protein/ml using a Pierce BCA assay (Thermo Scientific). When used, macrophages were co incubated with 20hr apoptotic neutrophils at an MOI of 10:1(healthy donors) and 15:2 (COPD donors) for 90min prior to vigorous washing for removal of any non-internalised neutrophils. In a separate experiment, to explore the nature of the increase in Spare Respiratory Capacity following co-incubation with

apoptotic neutrophils, healthy AM were co-incubated with apoptotic neutrophils or pre-treated with known M2 stimuli (E2,E3). Cells were pre-treated with 20ng/ml IL-4 (68-8780-63, eBioscience) and 20ng/ml IL-13 (571102, BioLegend) or 20ng/ml IL-10 (217-IL, R&D) for 16hrs prior to running seahorse assays.

Glycolytic Stress Test	*Amount of media added to vial:*
Vial 1 : Glucose	3000µl
Vial 2: Oligomycin	720µl
Vial 3 : 2DG	3000µl
Mitochondrial Stress Test	
Vial 1: Oligomycin	630µl. Then 225µl of vial was added to 1.275ml media.
Vial 2:FCCP	720µl. Then 300µl of vial added to 1.275ml media.
Vial 3: Rotenone & Antimycin	540µl. Then 300µl of vial added to 1.2ml media.

RNA isolation and quantification: RNA was isolated from macrophages (approximately 600,000 cells per sample) or THP-1 cells (approximately 1x10^6 cells per sample) using the mirVana total RNA isolation protocol (Ambion, Thermo Fisher),

DNase treated and reverse transcribed using AMV reverse transcriptase with random primers (Promega). Gene expression was analyzed using predesigned qPCR Primer/Probe assays and Prime Time Gene Expression Mastermix (IDT). Genes of interest were normalized to β actin expression.

Transcriptomic Analysis: ***(A)*** *COPD AM +/- KI-696 vs healthy AM +/- KI-696 data set:* RNA was extracted from Alveolar Macrophages and Total RNA-Sequencing performed by the Edinburgh Clinical Research Facility Genomics Core, Edinburgh UK. Analysis was performed by Thomson Bioinformatics as detailed : FastQ files were mapped to the reference genome (UCSC hg19) using the STAR alignment tool (STAR 2.6.1a) with filters applied allowing no more than 5 mismatches per read, read QC scores above a defined normalized threshold score of 0.66 and multimapping alignment scores no greater than 1. Following mapping overall metrics were analyzed to assess the quality of each run (i.e. % reads passing QC thresholds following removal of abundant RNA reads, median coverage uniformity statistics and comparative genomic alignment distribution analyses). Transcripts Per Million (TPM) scores were calculated for each gene across all samples. Differentially expressed (DE) genes were defined as genes displaying greater than Log_2 1.5 fold change and with P-values <0.05 between two sample cohorts (E4,E5,E6).

(B) COPD AM and Healthy Donor AM +/- *S. pneumoniae*: This data set was previously published by our group, with details outlined in the manuscript (E6). In brief , AM from Healthy and COPD donors were co-incubated with *D39 Streptococcus pneumoniae* for 6 hours before vigorous washing. RNA was then extracted and hybridized onto the Affymetrix HG-U133 plus 2.0 Array. The transcriptomic data is available online in the

ArrayExpress database at EMBL-EAI www.ebi.ac.uk/arrayexpress, under accession number EMTAB-6491. This data set was then independently re analysed by our lab group, specifically targeting metabolism-related changes in transcription.

HPLC-MS analysis of metabolite abundance: Samples were generated by harvesting approximately 600,000 AM, using 300μl of Methanol per sample, on ice. Samples were stored for at least 24 hours in -80 C before being thawed on ice and spun at 10,000G for 10 min at 4°C. Supernatant was removed for analysis using Dionex UltiMate 3000 LC System (Thermo Scientific) coupled to a Q Exactive Orbitrap mass spectrometer (Thermo Scientific) operated in negative mode. Data collection was performed using Xcalibur software (Thermo Scientific) verified by manual assessment of each sample and metabolite. Samples were normalized to protein content by adding 200μl of 200mM NaOH to each pellet. Samples were then boiled at 95 C for 20min, cooled on ice and spun down for 10min at 400G. Protein content was measured using a Pierce BCA assay (Thermo Scientific). For 13C tracing experiments, differentiated THP-1 cells were cultured in the presence of 5.5 mM U-13C glucose for 6 hours. Relative levels of isotopologue abundance and 13C incorporation into lactate were measured as above and compared to unlabelled (U-12C glucose) controls. Percentage labelling is expressed as the percentage of 13C isotopologue of the total lactate abundance.

Generation of proteomic samples: Alveolar macrophages were isolated from BAL fluid as detailed above and cultured for 3 days with daily washes prior to harvesting. Cells were pelleted at 400G for 8 minutes , supernatant discarded and pellets flash frozen. Protein extracts had the following added; SDS to a final concentration of 5%,

TCEP to a final concentration of 10mM and TEAB to a final concentration of 50mM. Samples were incubated at 95°C for 5 minutes before sonicating for 15 cycles of 30 seconds each using a BioRuptor (Diagenode). Proteins were alkylated in the dark for 1 hour by the addition of IAA at 20mM. Protein lysates were prepared for mass spectrometry using s-trap mini columns according to the manufactures instructions (Protifi). In summary, for each sample 200 mg of protein was loaded onto a s-trap mini column. Captured protein was washed 5 times with 400 ml of wash buffer (90% methanol with 100 mM TEAB, pH 7.1). Proteins were digested by the addition of 10 mg of trypsin to each sample in 50 mM ammonium bicarbonate. Samples were digested for 2 hours at 47 °C. Once digestion was complete, peptides were eluted with 80 ml of 50 mM ammonium bicarbonate followed by 80 ml of 0.2 % formic acid and lastly with the addition of 80 ml 50 % acetonitrile with 0.2 % formic acid. After the addition of each elution buffer, columns were centrifuged at 4000 g for 1 minute and the flow through collected. Eluted peptides were dried by speedvac and suspended in 1 % formic acid before quantification using the CBQCA assay (Invitrogen).

Proteomic Mass spectrometry analysis: For each sample 1.5 mg of peptide was analysed by data independent acquisition (DIA). Peptides were injected onto a nanoscale C18 reverse-phase chromatography system (UltiMate 3000 RSLC nano, Thermo Scientific) and electrosprayed into an Orbitrap Exploris 480 Mass Spectrometer (Thermo Fisher). For liquid chromatography the following buffers were used: buffer A {(0.1% formic acid in Milli-Q water (v/v)} and buffer B {(80% acetonitrile and 0.1% formic acid in Milli-Q water (v/v)}. Samples were loaded at 10 µL/min onto a trap column (100 µm × 2 cm, PepMap nanoViper C18 column, 5 µm, 100 Å, Thermo Scientific)

equilibrated in 0.1% trifluoroacetic acid (TFA). The trap column was washed for 3 min at the same flow rate with 0.1% TFA then switched in-line with a Thermo Scientific, resolving C18 column (75 µm × 50 cm, PepMap RSLC C18 column, 2 µm, 100 Å). Peptides were eluted from the column at a constant flow rate of 300 nl/min with a linear gradient from 3% buffer B to 6% buffer B in 5 min, then from 6% buffer B to 35% buffer B in 115 min, and finally to 80% buffer B within 7 min. The column was then washed with 80% buffer B for 4 min and re-equilibrated in 3% buffer B for 15 min. Two blanks were run between each sample to reduce carry-over. The column was kept at a constant temperature of 50°C. The data was acquired using an easy spray source operated in positive mode with spray voltage at 2.445 kV, and the ion transfer tube temperature at 250oC. The MS was operated in DIA mode. A scan cycle comprised a full MS scan (m/z range from 350-1650), with RF lens at 40%, AGC target set to custom, normalised AGC target at 300%, maximum injection time mode set to custom, maximum injection time at 20 ms, microscan set to 1 and source fragmentation disabled. MS survey scan was followed by MS/MS DIA scan events using the following parameters: multiplex ions set to false, collision energy mode set to stepped, collision energy type set to normalized, HCD collision energies set to 25.5, 27 and 30%, orbitrap resolution 30000, first mass 200, RF lens 40%, AGC target set to custom, normalized AGC target 3000%, microscan set to 1 and maximum injection time 55 ms. Data for both MS scan and MS/MS DIA scan events were acquired in profile mode.

Analysis of proteomic DIA-MS data: Raw mass spec data files were searched using Spectronaut (Biognosys) version 16.0.220606.53000 using the directDIA function. The following search settings were used: minimum peptide length 7, maximum peptide

length 52, cleavage enzyme Trypsin, maximum missed cleavages 2, protein and peptide FDR was set at 0.01, profiling and cross run normalisation were disabled. Carbamidomethyl (C) was selected as a fixed modification while Acetyl (N-term), Deamidation (NQ) and Oxidation (M) were selected as variable modifications. Data were searched against a human database with isoforms from Uniprot release 2021 01. Estimated protein copy numbers and concentration were calculated using the proteomic ruler (E7) and Perseus (E8). The normalized intensity for each identified protein was calculated by dividing the individual protein intensity by the total intensity obtained per sample to correct for technical variances during data acquisition between samples, prior to determining significance on Peresus .

Immunostaining for microscopy: Healthy and COPD patient lung sections were prepared from paraffin-embedded blocks. The lung sections were stained with anti-CD68 (ab201340, Abcam), anti-ME1 (ab223761, Abcam) after deparaffinization and antigen retrieval. The following were used - TSA plus system amplification (NEL744B001KT, Perkin Elmer) and autofluorescence quenching with TrueView (Vector, SP-8400). The nuclei were stained with DAPI (422801, Sigma-Aldrich). Images were acquired using a Leica SP8 confocal microscope at 20x magnification.

***ME1* knockout in THP-1 cells**: CRISPR-Cas9 mutagenesis was used to create a polyclonal population of *ME1*-null THP-1 cells. First, a protospacer sequence encoding a single guide RNA targeting the protein-coding sequence in the first exon of *ME1* (5' ATGGGTGTGGCGGCGACGGG-3') was cloned into LentiCRISPRv2 (a gift from Feng Zhang; Addgene plasmid #52961) according to previously described protocol (E9). The

vector was packaged into lentivirus using the LV-Max Lentiviral Production System (Gibco) according to the manufacturer's instructions. THP-1 cells were transduced, at a multiplicity of infection of approximately 1, by spin infection at 1000g for 1 hour at 32°C. Puromycin was added to the medium after 48 hours, and the cells were cultured under puromycin selection for 7 days.

ME1 chemical inhibition of monocyte-derived macrophages: Healthy MDM were pre-treated with a chemical ME1 inhibitor (Medchem HY-124861) at 100μM or DMSO vehicle control for 16 hours prior to running a mitochondrial stress test on Seahorse to determine basal OCR/ECAR ratios, as described above. To determine the effect of ME1 inhibition on basal mROS production, healthy MDM at day 12-14 of culture were transferred to iBidi 8-well chamber slides and subsequently treated for 16hrs with 100μM ME1 inhibitor or DMSO vehicle control. To detect mitochondrial ROS, MDM were washed x2 in warm HBSS, and incubated with 2μM MitoSox Red reagent (Thermo Fisher Scientific) in warm phenol red-free RPMI for 20 minutes at 37˚C. Cells were then washed again x2 in HBSS, and imaged immediately using an Andor spinning disk confocal microscope 60x objective, 0.5μM intervals at 610nm wavelength. Z-stack images were processed in Imaris software, and mean fluorescence intensity of MitoSox Red was obtained from >180 cells per condition from a total of 3 different donors.

GSH:GSSG Quantification: Samples were generated using 2 x 10^6 cells of THP-1 EV and THP-1 *ME1* KO cells. GSH:GSSG μmol/l was determined via a colorimetric assay,

as per the manufacturer's protocol (Sigma-Aldrich 38185). A 1 in 4 dilution was used for calculating GSH concentration.

mtDNA:nDNA content: 2 ng of DNA extracted using DNeasy Blood & Tissue Kit (QIAGEN) were subjected to qPCR using primers for mitochondrial DNA (mtDNA) mtF3212 (5'CACCCAAGAACAGGGTTTGT3'), mtR3319 (5'TGGCCATGGGTATGTTGTTAA3') and for nuclear DNA (nDNA) 18SrRNA gene 18S1546F (5'TAGAGGGACAAGTGGCGTTC3'), 18S1650R (5'CGCTGAGCCAGTCAGTGT3'). The probes used were 5'6-FAM/ZEN-TTACCGGGCTCTGCCATCT 3'IBFQ and 5' HEX-AGCAATAACAGGTCTCTGATG 3' BHQ®-2 for mtDNA and nDNA, respectively.

NRF2 activation via KI-696: Alveolar and monocyte-derived macrophages or THP-1 *ME1* KO cells were treated for 16 hours with the highly specific NRF2 activator, KI-696 at 0.065 µM ,provided by GSK (E10). Cells were then washed and harvested for RNA isolation (Fig. 5A, Fig. 6A-E) , assessment of metabolite abundance via HPLC-MS (Fig. 5 B-F) or co-incubated with PKH26 labelled 20h apoptotic neutrophils and run on flow cytometry (Fig. 6 F-G, fig. E6E), as described in the main manuscript materials and methods. To assess if NRF2 mediated rescue of COPD macrophage required sustained presence of KI-696, COPD MDM were (A) treated for 16 hours with KI-696 at then washed and rested for a further 16 hours prior to co-incubation with PKH26 labelled 20 hour apoptotic neutrophils or (B) treated for 16 hours with KI-696 then washed and immediately co-incubated with apoptotic neutrophils for assessment on flow cytometry (fig. E6D).

Bronchoalveolar fluid assessment: Aliquots of Bronchoalveolar Lavage Fluid were generated during the isolation of alveolar macrophages as described above. All nutrient concentrations were determined using fluorometric/colorimetric plate-based assays, as per the manufacturer's protocol and the optimal dilution of samples was ascertained for each different analyte. Concentrations used were: Glucose (Biovision K606-100): 1in2 dilution, 10 minute incubation; Glutamine (Abcam 197011) 1in2 dilution, 60 minute incubation; Lactate (Abcam 65330) 1in2 dilution, 30 minute incubation, prior to measuring nutrient concentration.

Determining intracellular glycogen concentrations: Glycogen stores were measured in both Monocyte-Derived Macrophages (MDM) and Alveolar Macrophages (AM). Three wells of approximately 200,000 cells per well were combined per condition. Wells were rapidly washed with cold PBS on ice x 3 to remove media then scraped in 200μl H20 per well. Lysates were boiled at 100°C for 10 min then spun at 13,000G for 5 min at 4°C. The supernatant glycogen content was determined via a fluorometric plate based assay (Sigma-Aldrich MAK016-1KT) at a 1:2 dilution, as per the manufacturer's guidelines. The cell pellet was stored for protein quantification and consequent glycogen content protein normalisation, via a Pierce BCA assay (Thermo Scientific).

Measuring Glucose uptake via a 2NBDG Assay: Uptake of 2NBDG was measured as a surrogate for glucose uptake using a 2NBDG kit (Cayman Chemical 600470). Cells were incubated in glucose free medium (Gibco, 11879-020) for 90min. 250μl of 240μM proprietary glucose was added to each well for 60 min before adding the Cell Based assay buffer and incubation on ice for 10 min. Supernatant was aspirated and 100μl of

1:100 Infra-Red Fixed viability (Biolegend,423195) stain was added, incubated for 10 minutes, light protected, on a shaking platform. 200μl of cell Based Assay buffer was then added and the TC plate was incubated on ice for 10 min prior to analysis on an Attune NXT flow cytometer. Control wells were pre-treated for 24 hours with Apigenin (included in the kit) to inhibit 2NBDG uptake.

Supplementary References :

E1.Gupta N, Pinto LM, Morogan A, Bourbeau J. The COPD assessment test: a systematic review. *Eur Respir J*. 2014;44(4):873-884.

E2.Jha AK, Huang SC-C, Sergushichev A, et al. Network integration of parallel metabolic and transcriptional data reveals metabolic modules that regulate macrophage polarization. *Immunity*. 2015;42(3):419-430. doi:10.1016/j.immuni.2015.02.005.

E3.Zhang S, Weinberg S, DeBerge M, et al. Efferocytosis Fuels Requirements of Fatty Acid Oxidation and the Electron Transport Chain to Polarize Macrophages for Tissue Repair. *Cell Metabolism*. 2019;29(2):443-456.e445. doi:10.1016/j.cmet.2018.12.004.

E4.Smith LC, Venosa A, Gow AJ, Laskin JD, Laskin DL. Transcriptional profiling of lung macrophages during pulmonary injury induced by nitrogen mustard. Ann N Y Acad Sci. 2020 Nov;1480(1):146-154. doi: 10.1111/nyas.14444.

E5.Shen L, Zhou K, Liu H, Yang J, Huang S, Yu F, Huang D. Prediction of Mechanosensitive Genes in Vascular Endothelial Cells Under High Wall Shear Stress. Front Genet. 2022 Jan 11;12:796812. doi: 10.3389/fgene.2021.796812.

E6. Bewley MA, Budd RC, Ryan E, et al. Opsonic Phagocytosis in Chronic Obstructive Pulmonary Disease Is Enhanced by NRF2 Agonists. *Am J Respir Crit Care Med*. 2018;198(6):739-750.

E7.Wiśniewski JR, Hein MY, Cox J, Mann M. A "proteomic ruler" for protein copy number and concentration estimation without spike-in standards. Mol Cell Proteomics. 2014 Dec;13(12):3497-506. doi: 10.1074/mcp.M113.037309.

E8.Tyanova, S., Temu, T., Sinitcyn, P. *et al.* The Perseus computational platform for comprehensive analysis of (prote)omics data. *Nat Methods* 13, 731–740 (2016). https://doi.org/10.1038/nmeth.3901

E9.Sanjana NE, Shalem O, Zhang F. Improved vectors and genome-wide libraries for CRISPR screening. *Nat Methods*. 2014;11(8):783-784.

E10. Davies TG, Wixted WE, Coyle JE, et al. Monoacidic Inhibitors of the Kelch-like ECH-Associated Protein 1: Nuclear Factor Erythroid 2-Related Factor 2 (KEAP1:NRF2) Protein-Protein Interaction with High Cell Potency Identified by Fragment-Based Discovery. *J Med Chem*. 2016;59(8):3991-4006. doi:10.1021/acs.jmedchem.6b00228

Supplementary Figures:

Figure E1. Age does not influence macrophage phagocytosis and efferocytosis.

Figure E2. COPD macrophages display equivalent basal metabolic rates coupled with a loss of metabolic plasticity, that is not attributable to a polarisation state.

Figure E3. Substrate availability and mitochondrial function does not differ between COPD and healthy donors.

Figure E4. Transcript expression of *ME1* in control and *ME1* knockout THP-1 cells.

Figure E5. Survey of proteomic markers does not support a senescence switch in COPD AM.

Figure E6. Protein abundance of NRF2 dependent and independent scavenger markers is reduced in COPD. NRF2 mediated pharmacological rescue of efferocytosis in COPD MDM requires sustained presence of the NRF2 agonist, KI-696, and the presence of ME1.

Figure E1. Age does not influence macrophage phagocytosis and efferocytosis. Correlation of bacterial internalization rates (A, n=19) and efferocytosis rates (B, n=28) with macrophage donor age. Data represents individual values. Pearson's correlation coefficient (r) and p values shown.

Figure E2. COPD macrophages display equivalent basal metabolic rates coupled with a loss of metabolic plasticity, that is not attributable to a polarisation state. (A-D) Glycolytic (A,C) and mitochondrial (B,D) stress testing was performed in COPD (circles) and healthy (squares) donor AM and MDM (A, HC n=6, COPD n=10, B, HC n=9, COPD n=12; C, n=5, D, n=7) Open symbols represent current smokers. COPD Smoker vs Ex-smoker for (A) P value=0.06, (B) P value= 0.41, (C) P value= 0.09. (E-F) Healthy and COPD donor macrophages were co incubated for 90 min with 20 h apoptotic neutrophils (+AN) prior to performing assays. Spare respiratory capacity (E, HC n=8, COPD n=6) and glycolytic reserve (F, HC n=5, COPD n=3) were determined by Seahorse analysis. (G) Healthy Donor AM were co incubated with 20h apoptotic neutrophils or treated for 16h with M2 polarizing stimuli 20ng/ml IL-10 or IL-4&13 (n=3). (H) Protein abundance of M1 and M2 Markers , as detected by data-independent acquisition MS proteomic analysis , in COPD relative to Healthy Non Smoker AM (diamonds) and COPD relative to "Healthy Smoker" AM (triangles). COPD AM n= 7, HNS and HS n= 5. Data represents individual values and mean ± SEM. Significance was determined by (A,B,D) unpaired t-test, (C) Mann-Whitney U Test, (E, F, G) two-way

ANOVA with Tukey's multiple comparisons. *P ≤0.05. ns= not significant . Apoptotic Neutrophil (AN) MOI=10:2 Healthy AM; MOI 15:2 COPD AM.

Figure E3. Substrate availability and mitochondrial function does not differ between COPD and healthy donors. (A) Relative protein abundance of mitochondrial fusion and fission proteins in COPD AM relative to Healthy Non-Smoker (HNS) and "Healthy Smoker" (HS) AM. COPD AM n= 7, HNS and HS n= 5. Protein abundance measured by data-independent acquisition MS proteomic analysis. (B-D) Colorimetric assays were used to determine glutamine (B, HC n=9, COPD n=8), Glucose (C, HC n=8, COPD n=9) and lactate concentration (D, n=8) in Bronchoalveolar Lavage (BAL) fluid from COPD (circles) and healthy(squares) donors. (E) Glucose uptake was measured in MDM via a 2-NBDG assay and analysed via flow cytometry, n=7. Uptake rates were normalised to median cell fluorescence of unstained cells. (F-G)The fold change increase in ECAR rates following glucose injection was calculated for healthy and COPD AM (E, HC n=5, COPD n=7) and MDM (F, n=5, No Glucose [NG] conditions n=4). Prior glucose deprivation for 16h was used as a positive control. (H) Glycogen abundance was measured in healthy (n=5) and COPD (n=6) AM and MDM using a colorimetric assay. Data represents individual values and mean ± SEM. P values calculated by (A) 2-way ANOVA with Tukey's multiple comparisons, (B-E) unpaired t-test, (F,H) Mann Whitney U test and (G) Kruskal Wallis test with Dunn's multiple comparisons. * P ≤0.05, **P ≤0.01, ns= not significant. MFI= Median Fluorescence Intensity, FMO =Fluorescence minus one, unstained cells. NG= No Glucose.

Figure E4. Transcript expression of malic enzyme1 (ME1) in control and *ME1* knockout THP-1 cells. *ME1* was deleted in the THP1 macrophage cell line using CRISPR cas9 mutagenesis. Control (EV) and *ME1* knockout (ME1 KO) THP1 cells were lysed and qPCR analysis of cDNA performed with data normalized to β- Actin (ACTB) expression, n= 3. Data represents individual values and mean ± SEM. P value calculated by paired t-test.

Figure E5. Survey of proteomic markers does not support a senescence switch in COPD AM. (A-F) Relative protein abundance in COPD, Healthy non-smoker (HNS) and "Healthy Smoker" (HS) AM of detected makers of senescence. COPD AM n= 7, HNS and HS n= 5. Protein abundance determined by data-acquisition independent MS proteomic analysis. Data represents individual values and mean ± SEM. P values calculated by (A-D) Kruskal-Wallis test with Dunn's multiple comparisons. * P ≤0.05, ns= not significant.

Figure E6. Protein abundance of NRF2 dependent and independent scavenger markers is reduced in COPD. NRF2 mediated pharmacological rescue of efferocytosis in COPD MDM requires sustained presence of the NRF2 agonist, KI-696, and the presence of ME1. (A-C) Protein abundance of the NRF2 dependent (MARCO) and NRF2 independent scavenger receptors (SRA1 and SRA6) is reduced in COPD AM relative to Healthy Non/Smoker AM (HNS,HS). COPD n= 7, HNS and HS n=5. Protein abundance was measured by data-independent acquisition MS proteomic analysis. (D) COPD MDM were treated for 16 hours with KI-696 or treated with KI-696 for 16 hours then washed and rested for 16 hours (KI-696 wash off) prior to co-incubation with PKH26 labelled 20h apoptotic neutrophils, n=4. (E) *ME1* knockout (*ME1* KO) THP1

macrophage cells were incubated for 16 hours in the presence or absence of KI-696 prior to co-incubation with PKH26 labelled 20h apoptotic neutrophils, n=7. Efferocytosis rates were measured via flow cytometry. Data represents individual values and mean ± SEM. P values calculated by (A-C) Kruskal-Wallis test with Dunn's multiple comparisons, (D) 2-way ANOVA with Dunnet's multiple comparisons and (E) paired t-test. * P ≤0.05, **P ≤0.01, ns= not significant.

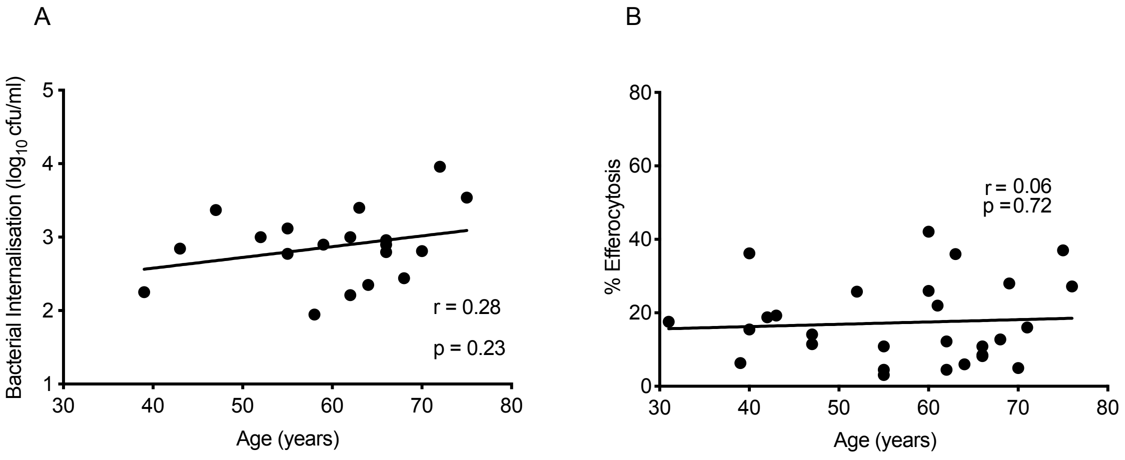
A
B
Bacterial Internalisation (log10 cfu/ml)
5
4
3
2
1
30
40
50
60
70
80
Age (years)
r = 0.28
p = 0.23
% Efferocytosis
80
60
40
20
0
r = 0.06
p =0.72

Figure E1:

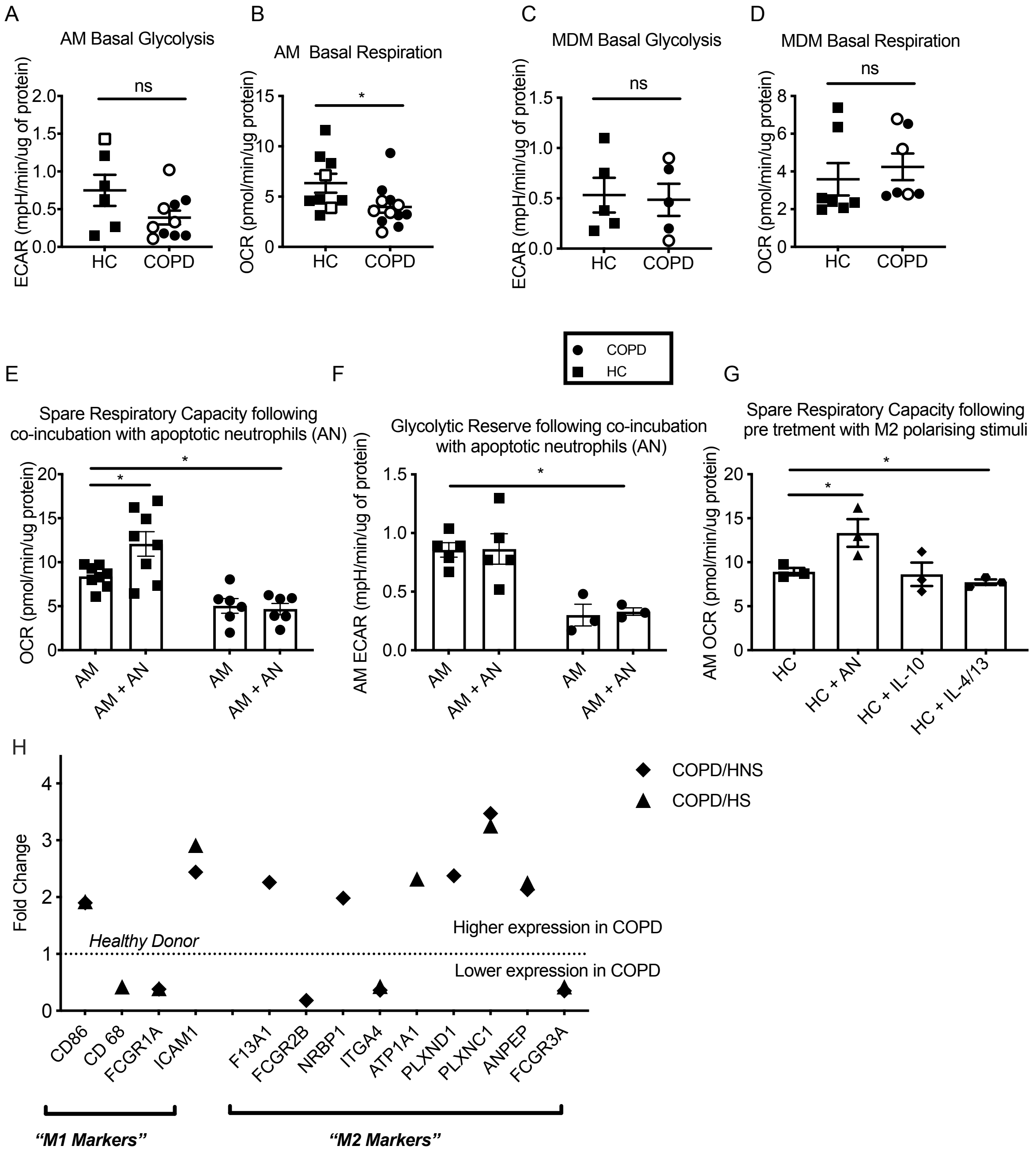

A
AM Basal Glycolysis
ECAR (mpH/min/ug of protein)
ns
HC
COPD
B
AM Basal Respiration
OCR (pmol/min/ug protein)
*
C
MDM Basal Glycolysis
D
MDM Basal Respiration
COPD
HC
E
Spare Respiratory Capacity following co-incubation with apoptotic neutrophils (AN)
AM
AM + AN
F
Glycolytic Reserve following co-incubation with apoptotic neutrophils (AN)
AM ECAR (mpH/min/ug of protein)
G
Spare Respiratory Capacity following pre tretment with M2 polarising stimuli
AM OCR (pmol/min/ug protein)
HC + AN
HC + IL-10
HC + IL-4/13
H
COPD/HNS
COPD/HS
Fold Change
Healthy Donor
Higher expression in COPD
Lower expression in COPD
CD86
CD 68
FCGR1A
ICAM1
F13A1
FCGR2B
NRBP1
ITGA4
ATP1A1
PLXND1
PLXNC1
ANPEP
FCGR3A
"M1 Markers"
"M2 Markers"

Figure E2:

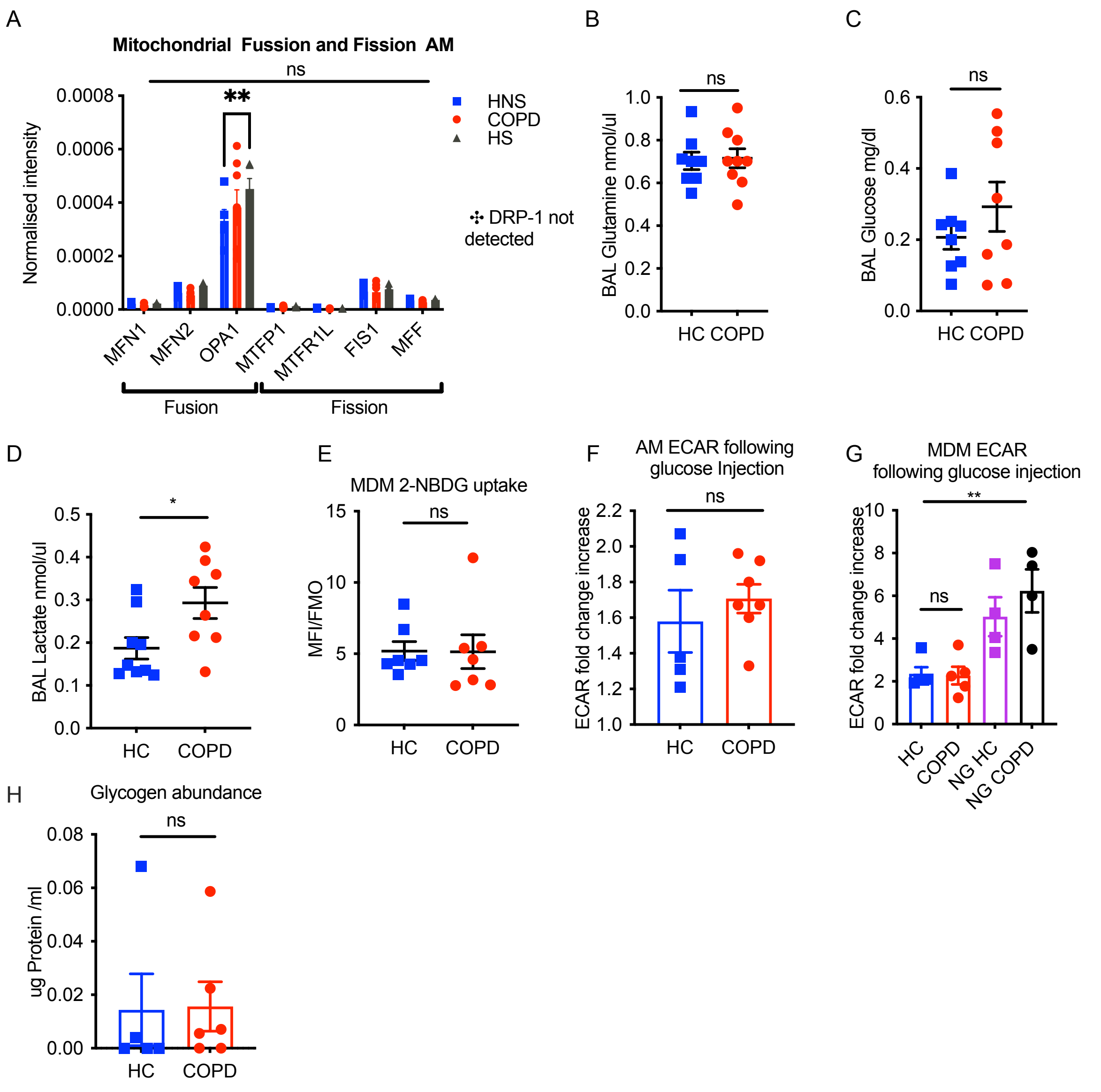
A
Mitochondrial Fussion and Fission AM
ns
**
Normalised intensity
0.0008
0.0006
0.0004
0.0002
0.0000
HNS
COPD
HS
DRP-1 not detected
MFN1
MFN2
OPA1
MTFP1
MTFR1L
FIS1
MFF
Fusion
Fission
B
ns
BAL Glutamine nmol/ul
1.0
0.8
0.6
0.4
0.2
0.0
HC COPD
C
ns
BAL Glucose mg/dl
0.6
0.4
0.2
0.0
HC COPD
D
*
BAL Lactate nmol/ul
0.5
0.4
0.3
0.2
0.1
0.0
HC
COPD
E
MDM 2-NBDG uptake
ns
MFI/FMO
15
10
5
0
HC
COPD
F
AM ECAR following glucose Injection
ns
ECAR fold change increase
2.2
2.0
1.8
1.6
1.4
1.2
1.0
HC
COPD
G
MDM ECAR following glucose injection
**
ns
ECAR fold change increase
10
8
6
4
2
0
HC
COPD
NG HC
NG COPD
H
Glycogen abundance
ns
ug Protein /ml
0.08
0.06
0.04
0.02
0.00
HC
COPD

Figure E3:

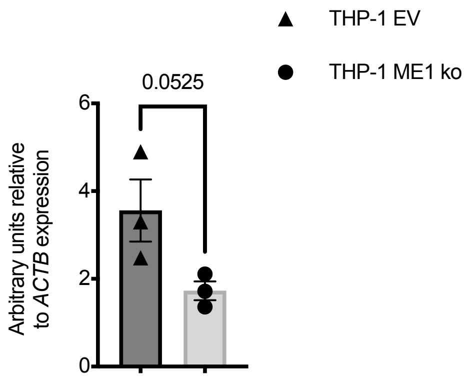
THP-1 EV
THP-1 ME1 ko
0.0525
Arbitrary units relative to *ACTB* expression
6
4
2
0

Figure E4:

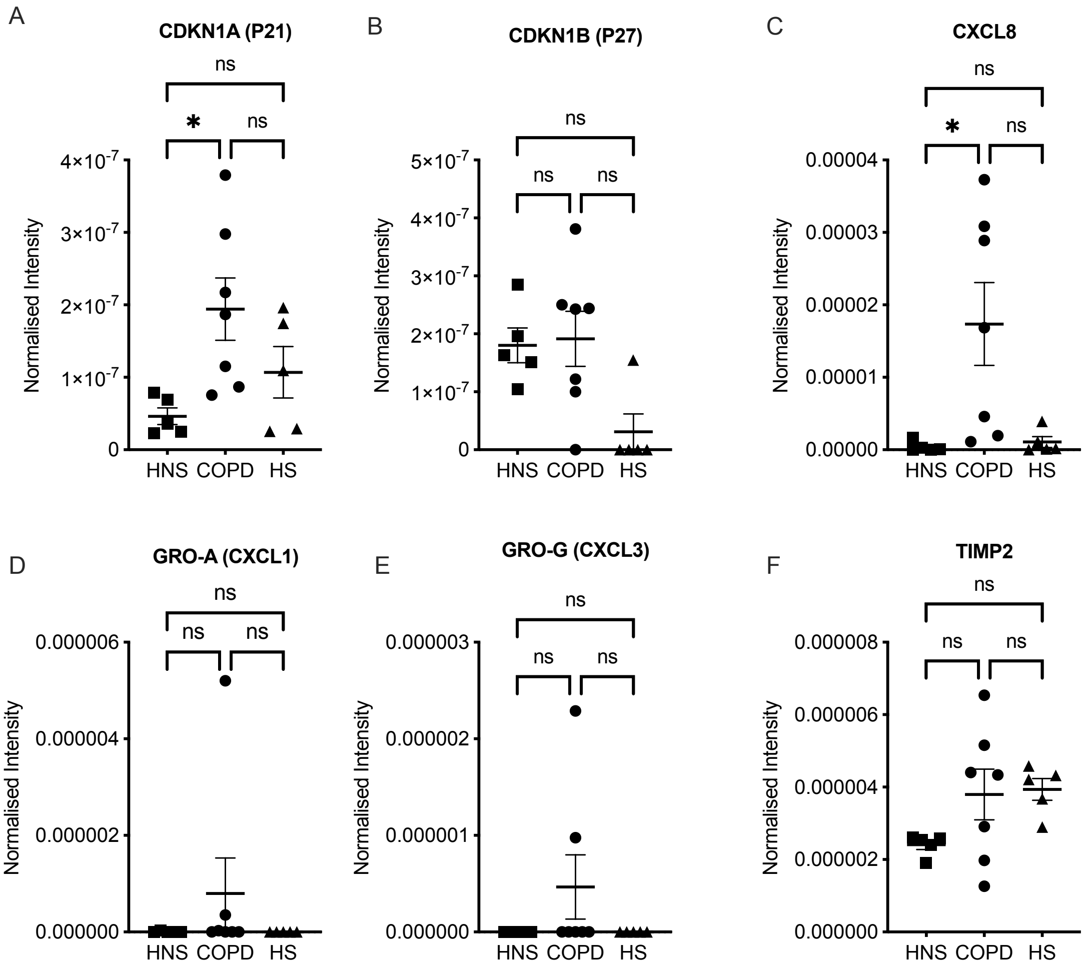
A
CDKN1A (P21)
B
CDKN1B (P27)
C
CXCL8
D
GRO-A (CXCL1)
E
GRO-G (CXCL3)
F
TIMP2
Normalised Intensity
HNS COPD HS
ns
*

Figure E5:

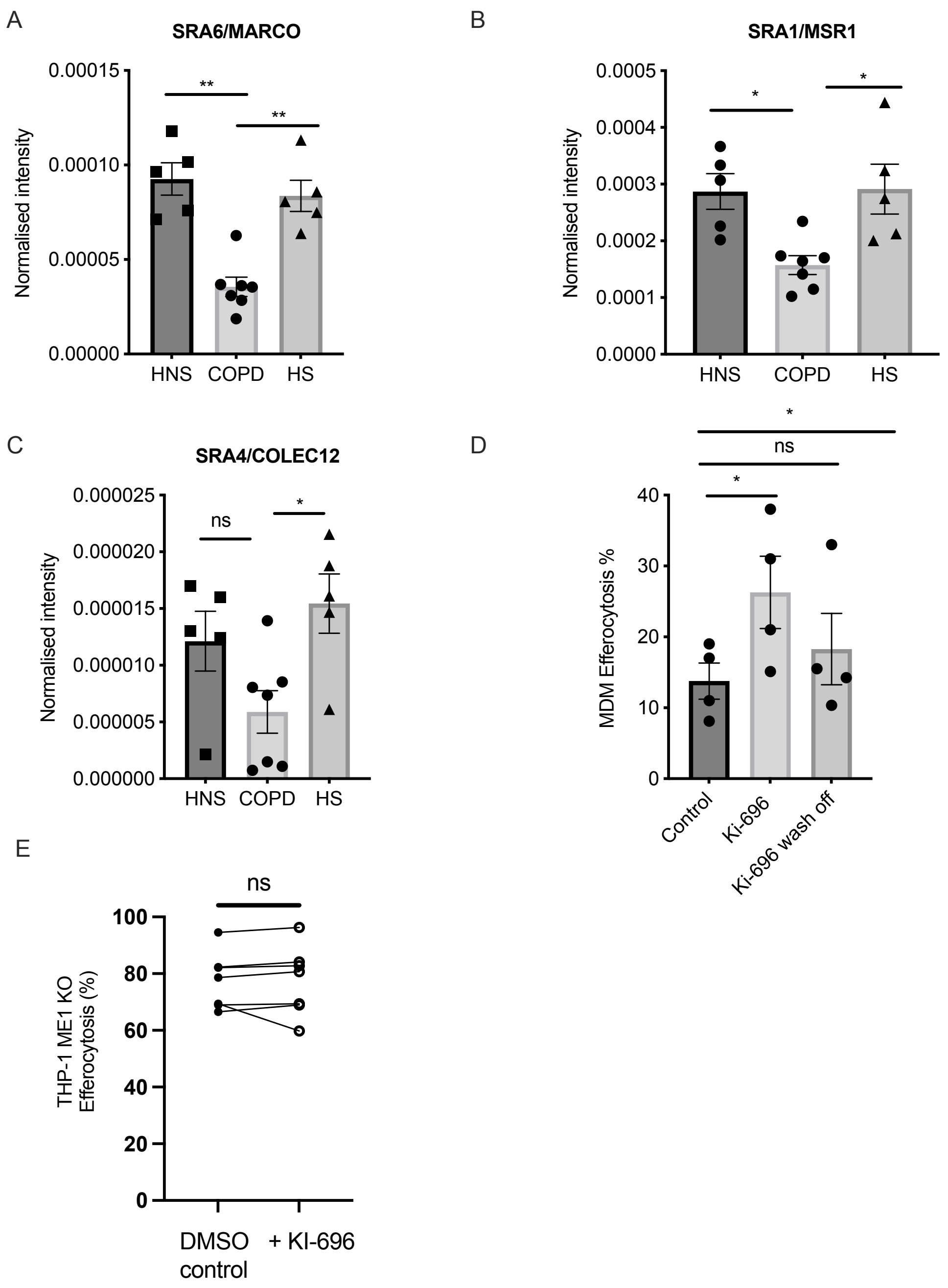
A
SRA6/MARCO
Normalised intensity
0.00015
0.00010
0.00005
0.00000
**
**
HNS
COPD
HS
B
SRA1/MSR1
Normalised intensity
0.0005
0.0004
0.0003
0.0002
0.0001
0.0000
*
*
HNS
COPD
HS
C
SRA4/COLEC12
Normalised intensity
0.000025
0.000020
0.000015
0.000010
0.000005
0.000000
ns
*
HNS
COPD
HS
D
*
ns
*
MDM Efferocytosis %
40
30
20
10
0
Control
Ki-696
Ki-696 wash off
E
ns
THP-1 ME1 KO Efferocytosis (%)
100
80
60
40
20
0
DMSO control
+ KI-696

Figure E6:
